# Supplementary material for: An extracellular receptor tyrosine kinase motif orchestrating intracellular STAT activation
Source: Nat Commun. 2022 Nov 14;13:6953. doi: 10.1038/s41467-022-34539-4 (PMC9663514; doi:10.1038/s41467-022-34539-4)
Supplement: Supplementary file 1 — Supplementary Information [file 41467_2022_34539_MOESM1_ESM.pdf]

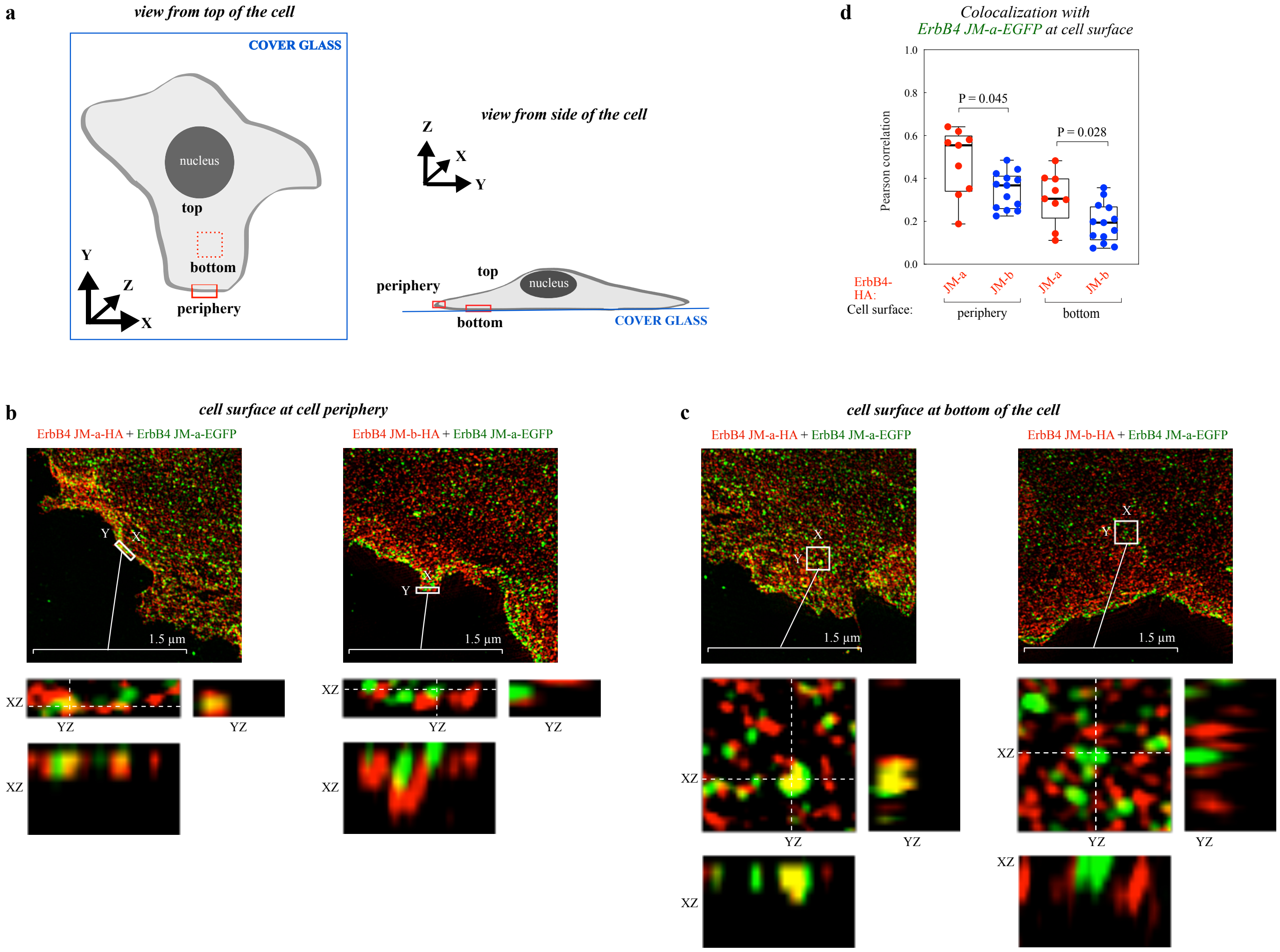

Supplementary Figure 1. Localization of ErbB4 JM isoforms at different compartments of the cell surface.
a: Schematic representation of the imaging strategy.
b-d: SIM super-resolution immunofluorescence analysis of colocalization of HA-tagged (red) and EGFP-tagged (green) ErbB4 JM isoforms in COS-7 cells. The imaging was focused on areas representing cell surfaces in the lateral periphery of the cell (b) or in the in the bottom of the cell at the surface facing the cover glass on the culture plate (c). The white boxes highlight the regions of interest in the x-y plane that are magnified below. The white dashed lines correspond to the position of the x-z (below the magnified view) and y-z (right of the magnified view) projections in the x-y image. Panel d depicts quantification of co-localization of HA and EGFP epitope-derived signals at the different cell surfaces in the x-y plane where each dot represents the correlation of the signals in one cell. n=9-13 cells examined over 2 independent experiments.Two-tailed Mann-Whitney U test. In the boxplots the line represents the median, the box the interquartile range and whiskers the whole range of values. Source data are provided as a Source Data file.

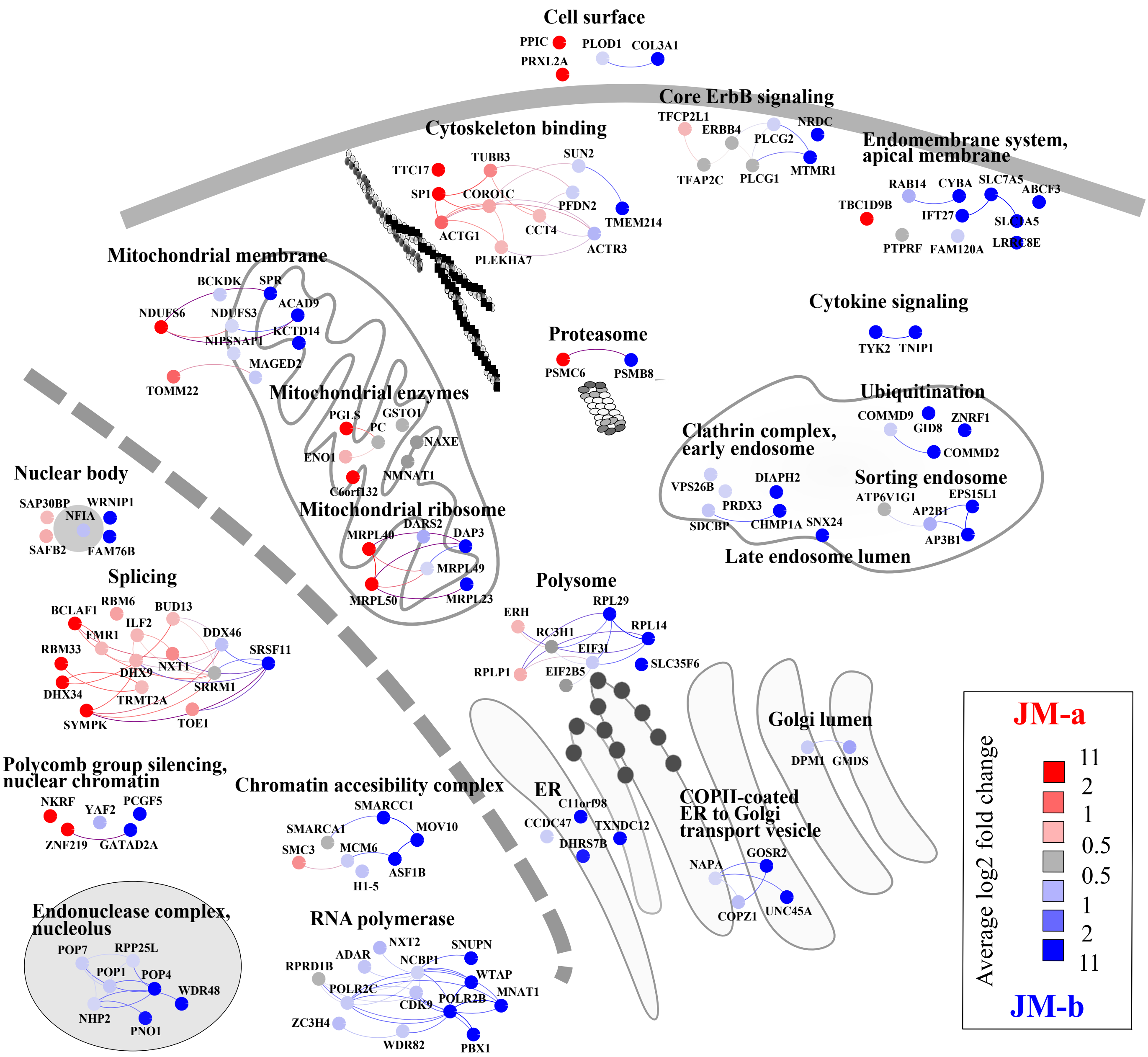

Supplementary Figure 2. ErbB4 JM isoforms associate with different signaling complexes. Modeled signaling complexes for ErbB4 JM-a and JM-b based on their mass spectrometry-derived interactomes in MDA-MB-468 cells expressing ErbB4 JM-a or ErbB4 JM-b. Colors indicate the preference of interaction with either JM-a or JM-b: red, interaction exclusively with JM-a; grey, equal interaction with JM-a and JM-b; blue, interaction exclusively with JM-b. The shade of color corresponds to the average log2 fold change against vector control cells in four replicate experiments. Only statistically significant interactors are included (adjusted *P*-value $\leq$ 0.05). The edges in the graphs represent experimentally determined protein-protein interactions as defined by STRING and PISQUIC databases. n=4 independent experiments. Source data are provided as a Source Data file.

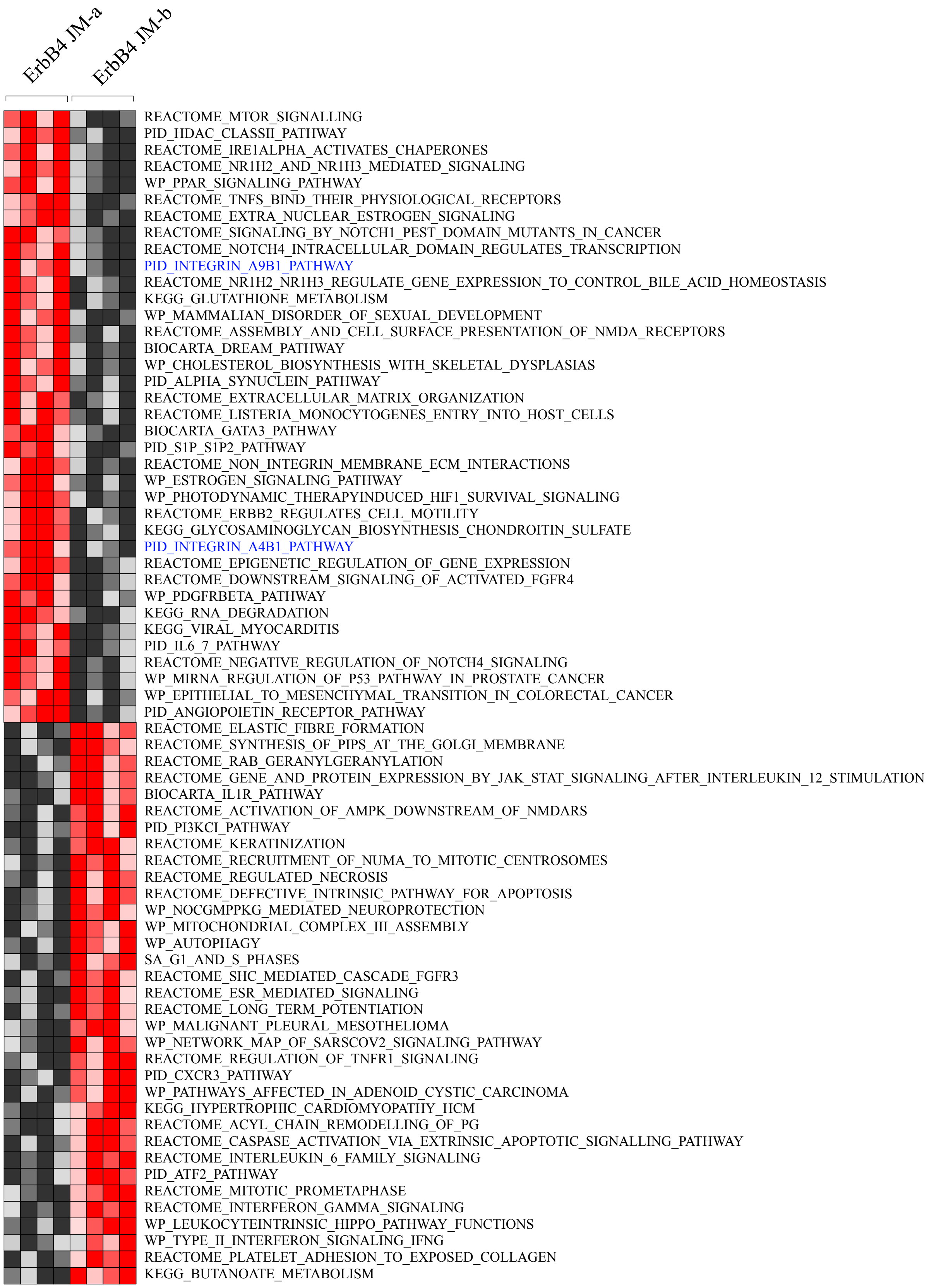

Supplementary Figure 3. **Pathway analysis of the mass-spectrometry derived interactomes of ErbB4 JM isoforms.**

Pathway annotations for interactomes of ErbB4 JM-a and JM-b in MDA-MB-468 cells were acquired from the Molecular Signatures database. Stochastic neighborhood embedding was utilized to estimate an expression value for each pathway in each sample. The differential expression of a pathway between ErbB4 JM-a and ErbB4 JM-b was estimated with two-tailed Mann-Whitney U-test. Only statistically differentially expressed pathways are shown (P-value < 0.05). The color in the heatmap corresponds to the pathway value: dark red, 100; white, 0; dark grey, -100. β1 integrin pathways are shown in blue. Source data are provided as a Source Data file.

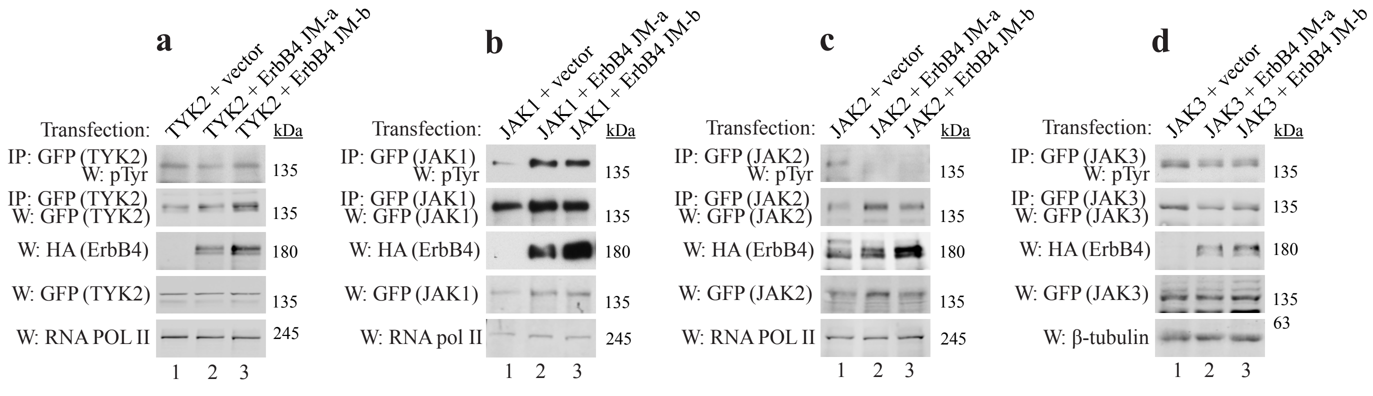

Supplementary Figure 4. JAK kinase phosphorylation in ErbB4-stimulated STAT5 signaling. Phosphorylation of JAK1 and JAK3 in MCF-7 cells overexpressing GFP-tagged TYK2 (a), JAK1 (b), JAK2 (c), or JAK3 (d), and HA-tagged ErbB4 JM-a or JM-b. Cells were stimulated with NRG-1. Representative blots of n=2 independent experiments. Source data are provided as a Source Data file.

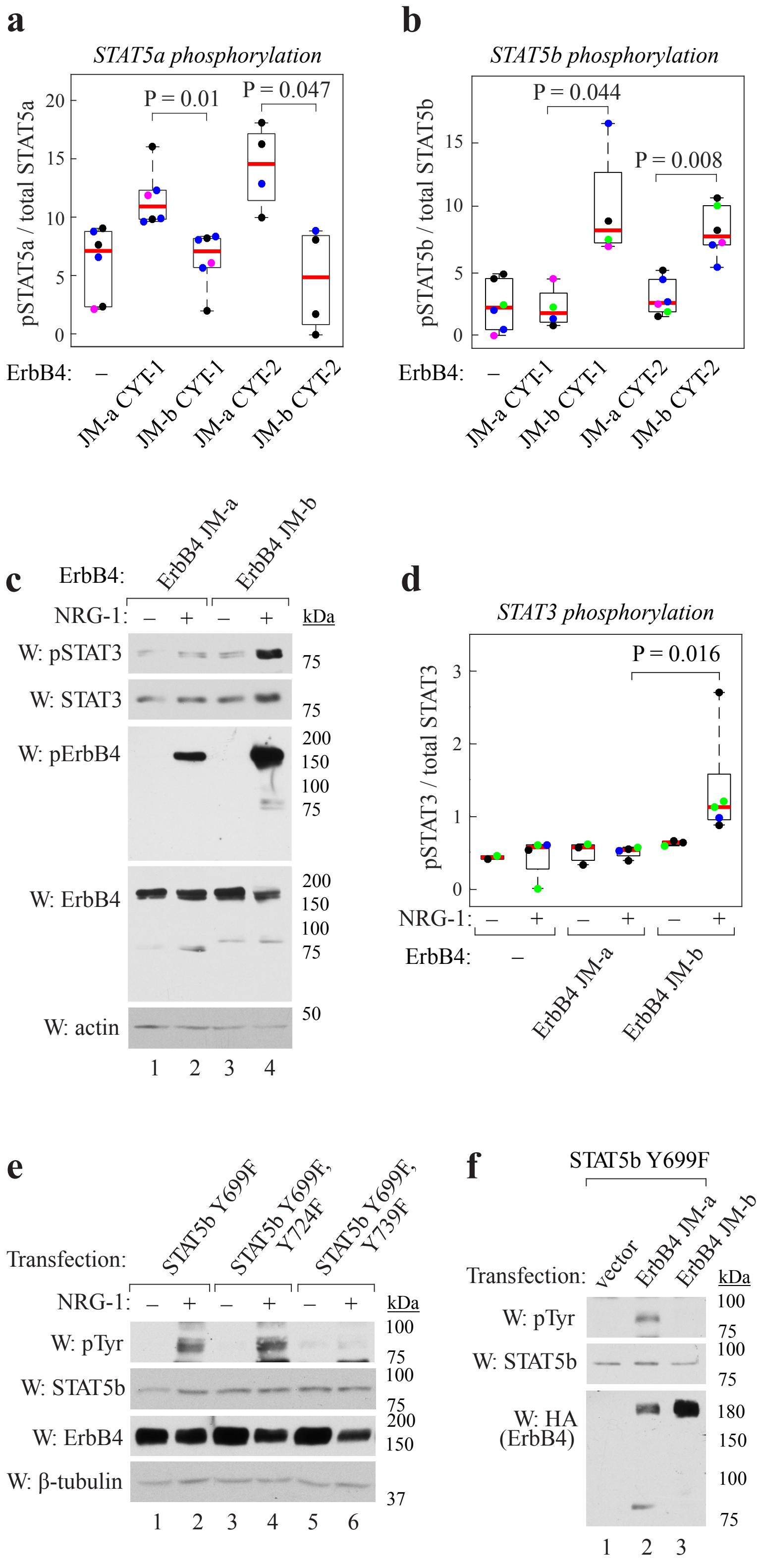

Supplementary Figure 5. STAT phosphorylation by ErbB4 JM isoforms.

a-b: Densitometric quantification of Western analyses of STAT5 phosphorylation (on Y694/699). Cells were transfected with constructs expressing ErbB4 JM isoforms in the context of two different cytoplasmic domains, CYT-1 and CYT-2. Black symbol, MDA-MB-468 cells; blue, HC11 cells; green, MCF-7 cells; magenta, COS-7 cells. n=4-6 independent experiments. Two-tailed Mann-Whitney U test.

c: Phosphorylation status of STAT3 (on Y705) in MDA-MB-468 cells expressing ErbB4 JM-a or ErbB4 JM-b. Cells were stimulated with NRG-1 where indicated.

d: Densitometric quantification of STAT3 Western analyses, such as shown in panel c. Black symbol, MDA-MB-468 cells; blue, HC11 cells; green, MCF-7 cells. n=5 independent experiments. Mack-Skillings two-way ANOVA.

e-f: Phosphorylation of the indicated STAT5b mutants in MCF-7 cells endogenously expressing ErbB4 JM-a (e) and in COS-7 cells overexpressing ErbB4 JM-a or ErbB4 JM-b (f). Phosphorylation was detected using a phosphotyrosine antibody not specific for a single phosphorylation site. Cells were stimulated with NRG-1 where indicated. Representative blots of n=3 independent experiments.

In the boxplots the line represents the median, the box the interquartile range and whiskers the whole range of values. Source data are provided as a Source Data file.

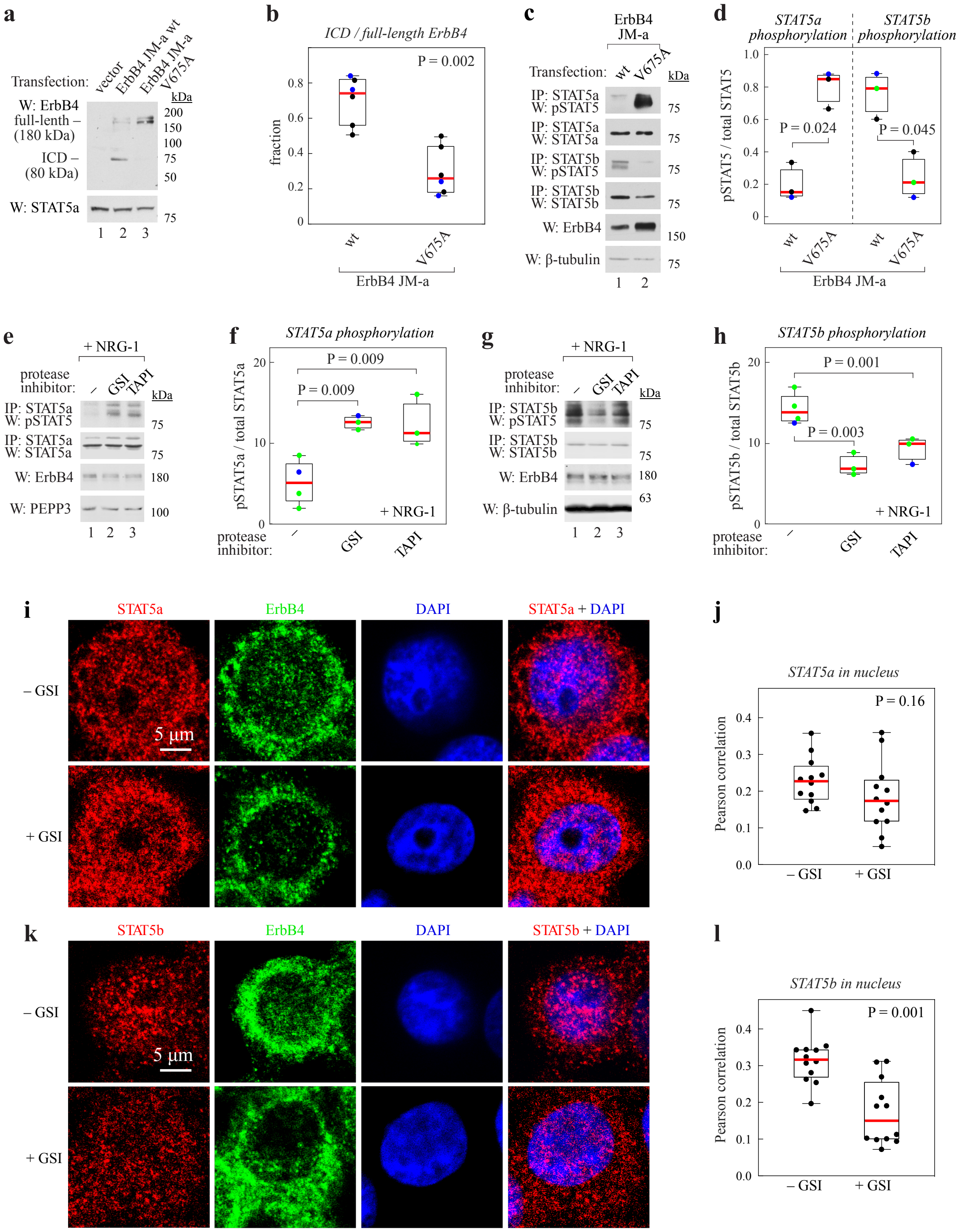

Supplementary Figure 6. ErbB4 cleavage and STAT5 activation.

a-b: Western analysis of the abundance of an 80 kD intracellular domain (ICD) fragment generated from the indicated ErbB4 JM-a constructs in MDA-MB-468 cells. Panel b depicts densitometric quantification of independent experiments. Black symbol, MDA-MB-468 cells; blue, HC11 cells. n=6 independent experiments. Two-tailed Mann-Whitney U test.

c: Phosphorylation of STAT5a and STAT5b (on Y694/699) in HC11 cells expressing wild-type (wt) or a gamma secretase cleavage-resistant mutant (V675A) form of ErbB4 JM-a.

d: Densitometric quantification of STAT5 Western analyses, such as shown in panel c. Black symbol, MDA-MB-468 cells; blue, HC11 cells; green, MCF-7 cells. n=3 independent experiments. Two-tailed T-test.

e-h: Phosphorylation of STAT5a and STAT5b (on Y694/699) in protease inhibitor-treated MCF-7 cells that endogenously express ErbB4 JM-a. Cells were stimulated with NRG-1 and ErbB4 cleavage inhibited by GSI IX or TAPI-0. Panels f and h depict densitometric quantification of independent experiments. Blue symbol, HC11 cells; green, MCF-7 cells. n=3 independent experiments. One-way ANOVA. Benjamini, Krieger and Yekutieli adjusted P-values.

i-l: Immunofluorescence analysis of nuclear localization of STAT5a (i,j) and STAT5b (k,l) in MDA-MB-468 cells expressing ErbB4 JM-a. Cells were treated with GSI IX or not. Confocal microscopy images (i,k) and quantification of the co-localization of the STAT5 subtype-specific signal and the chromatin stain DAPI (j,l) are shown. n=12 images analyzed over 2 independent experiments. Two-tailed T-test.

In the boxplots the line represents the median, the box the interquartile range and whiskers the whole range of values. Source data are provided as a Source Data file.

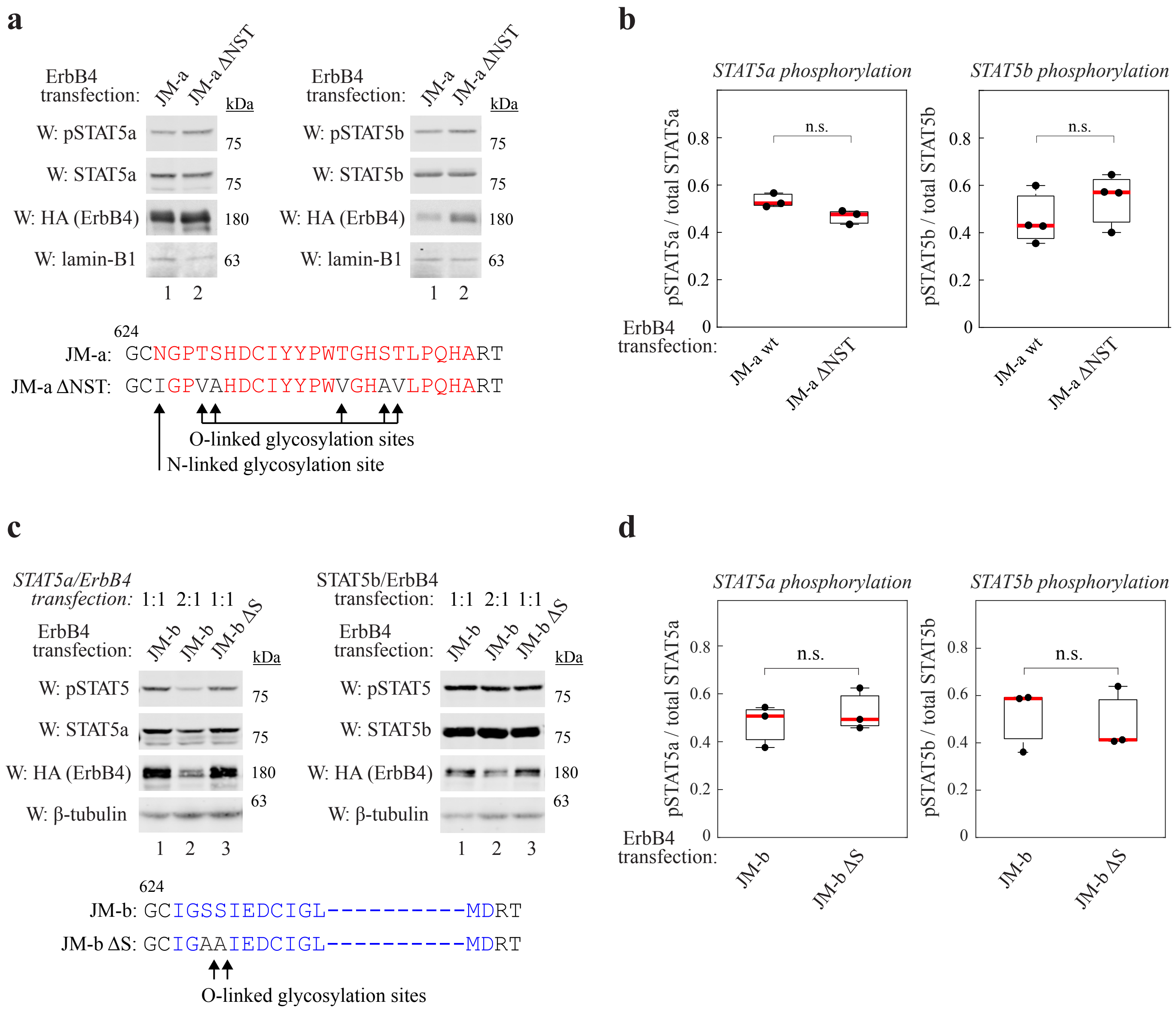

Supplementary Figure 7. ErbB4 glycosylation and STAT5 activation.
Phosphorylation of STAT5a and STAT5b (on Y694/699) in COS-7 cells expressing ErbB4 JM-a, ErbB4 JM-b or ErbB4 JM-a ΔNST or ErbB4 JM-b ΔS constructs with mutations at putative glycosylation sites. Sequence alignments of ErbB4 JM-a and ErbB4 JM-a ΔNST mutant (N626I/T629V/S630A/T639V/S642A/T643V), and of ErbB4 JM-b and ErbB4 JM-b ΔS mutant (S628A/S629A) are indicated. In panel c the plasmids encoding wild-type ErbB4 JM-b and STAT5b were co-transfected in two different plasmid DNA ratios (1:1 and 1:2 for ErbB4 JM-b and STAT5b, respectively). Panels b and d depict densitometric quantification of independent experiments. n.s., non-significant. n=3-4 independent experiments. Two-tailed T-test. In the boxplots the line represents the median, the box the interquartile range and whiskers the whole range of values. Source data are provided as a Source Data file.

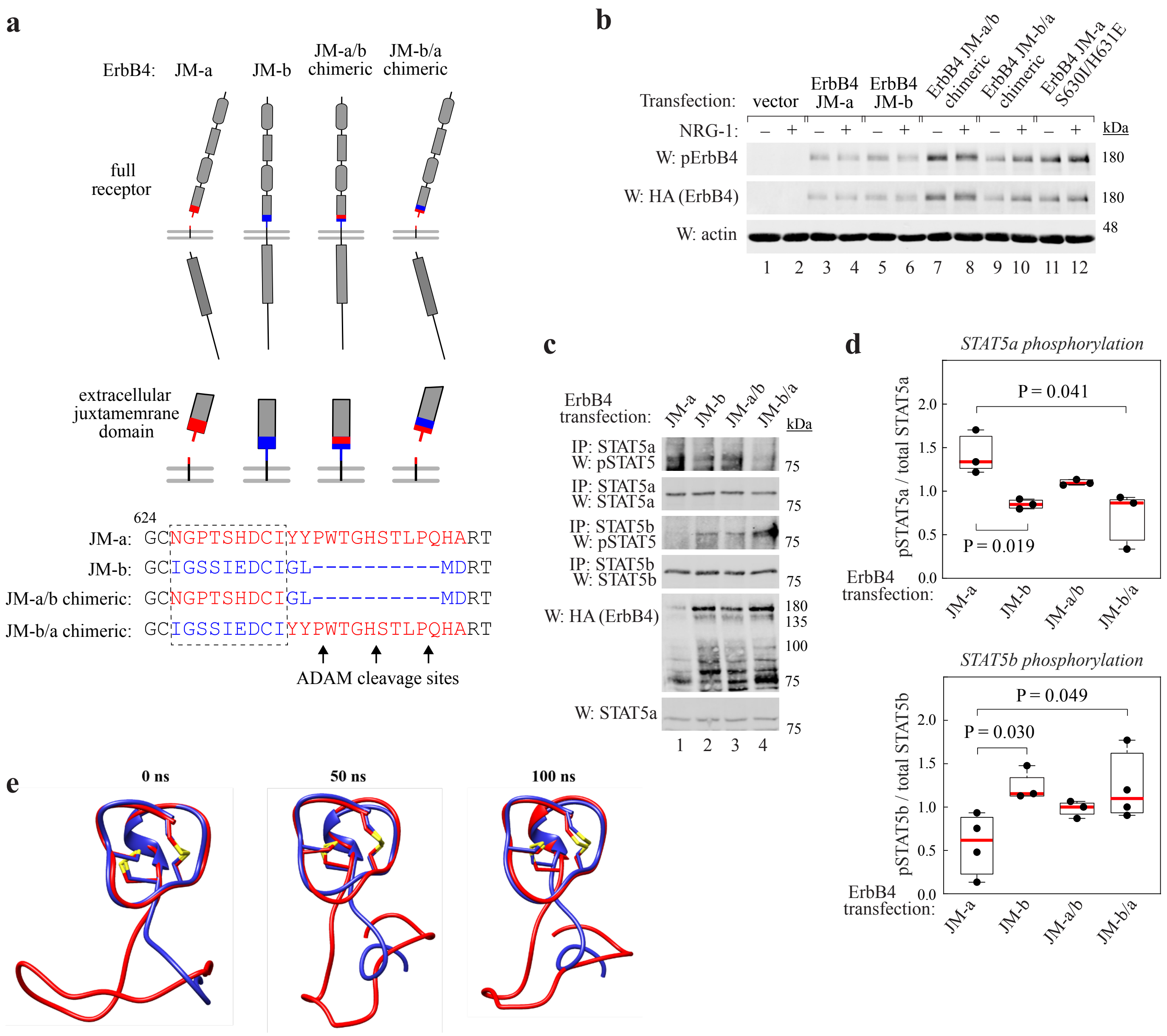

Supplementary Figure 8. ErbB4 eJM sequences regulating isoform-specific STAT5 activation.
a: A schematic presentation and sequence alignment of ErbB4 JM-a, ErbB4 JM-b, and chimeric ErbB4 JM mutants. ErbB4 JM chimeric mutants were created by exchanging the residues 626-634 (dashed box) between the JM-a and JM-b isoforms. The ADAM cleavage sites in the JM-a isoform are indicated.
b: Phosphorylation of ErbB4 in COS-7 cells overexpressing the indicated ErbB4 constructs. Cells were stimulated with NRG-1 where indicated. Representative blots of 2 independent experiments.
c-d: Phosphorylation of STAT5a and STAT5b (on Y694/699) in COS-7 cells expressing ErbB4 JM-a, ErbB4 JM-b, or the chimeric ErbB4 JM-a/b and ErbB4 JM-b/a constructs. Cells were stimulated with NRG-1. Densitometric quantification of independent experiments is shown in panel d. n=3-4 independent experiments. One-way ANOVA. Benjamini-Krieger and Yekutieli adjusted P-values. In the boxplots the line represents the median, the box the interquartile range and whiskers the whole range of values.
e: Sampled conformations of the C617-A648 region in ErbB4 JM-a (red) and JM-b (blue) isoforms during a 100 ns simulation. The disulfide constrained region (C617-C633) aligns well between the two isoforms in the conformations sampled at 0 ns, 50 ns and 100 ns simulation time.
Source data are provided as a Source Data file.

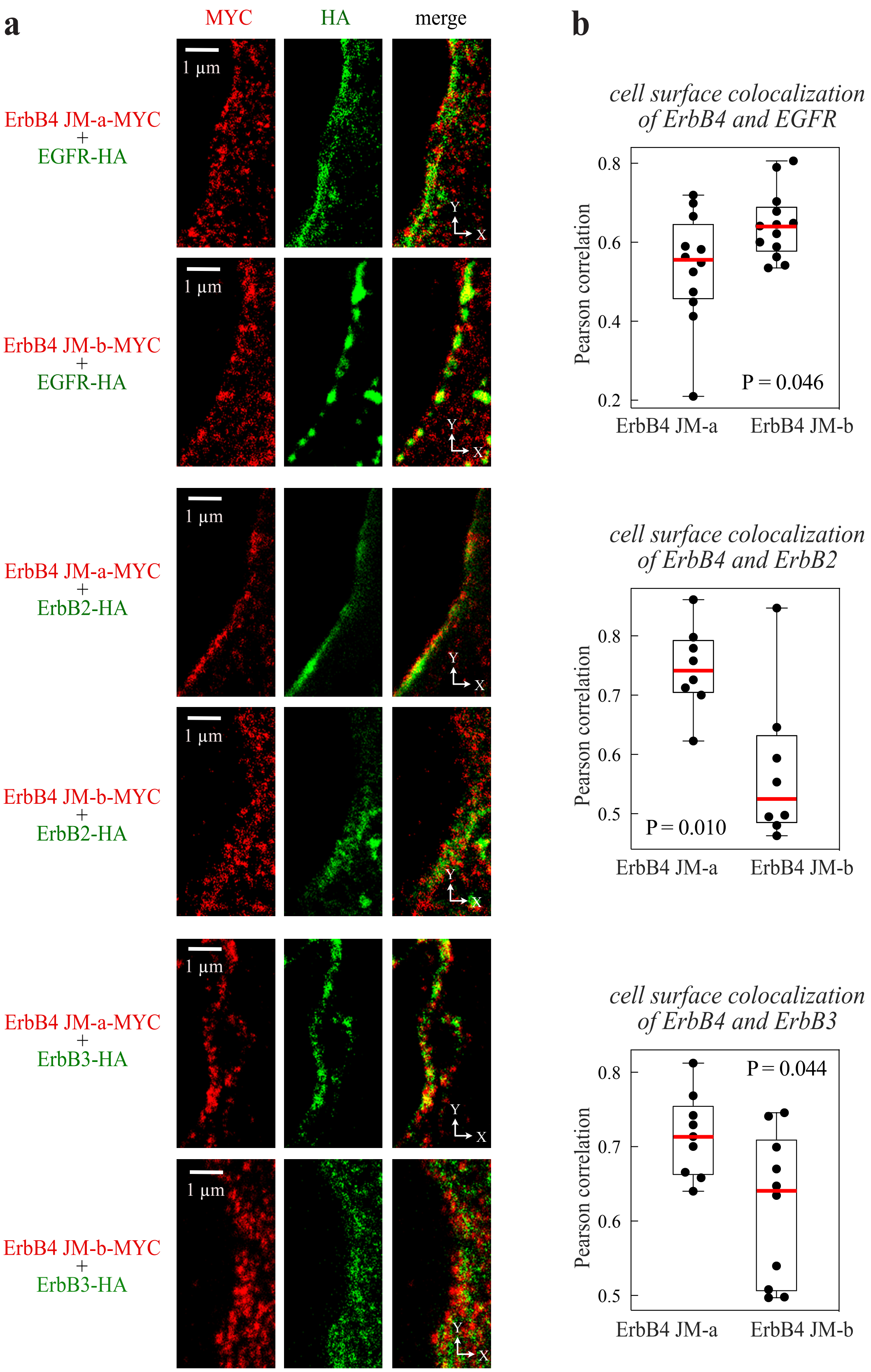

Supplementary Figure 9. Colocalization of other ErbB family members with ErbB4 JM-a and JM-b.
a: STED super-resolution immunofluorescence analysis of COS-7 cells expressing the indicated MYC-tagged (red) and HA-tagged (green) ErbB constructs. Regions of interest in the x-y plane are shown.
b: Quantification of co-localization of MYC- and HA-specific signals derived from the ErbB constructs as shown in panel A, where one dot represents the correlation of the signals in one cell. n=8-12 cells examined over 2 independent experiments. Mann-Whitney U test. In the boxplots the line represents the median, the box the interquartile range and whiskers the whole range of values. Source data are provided as a Source Data file.

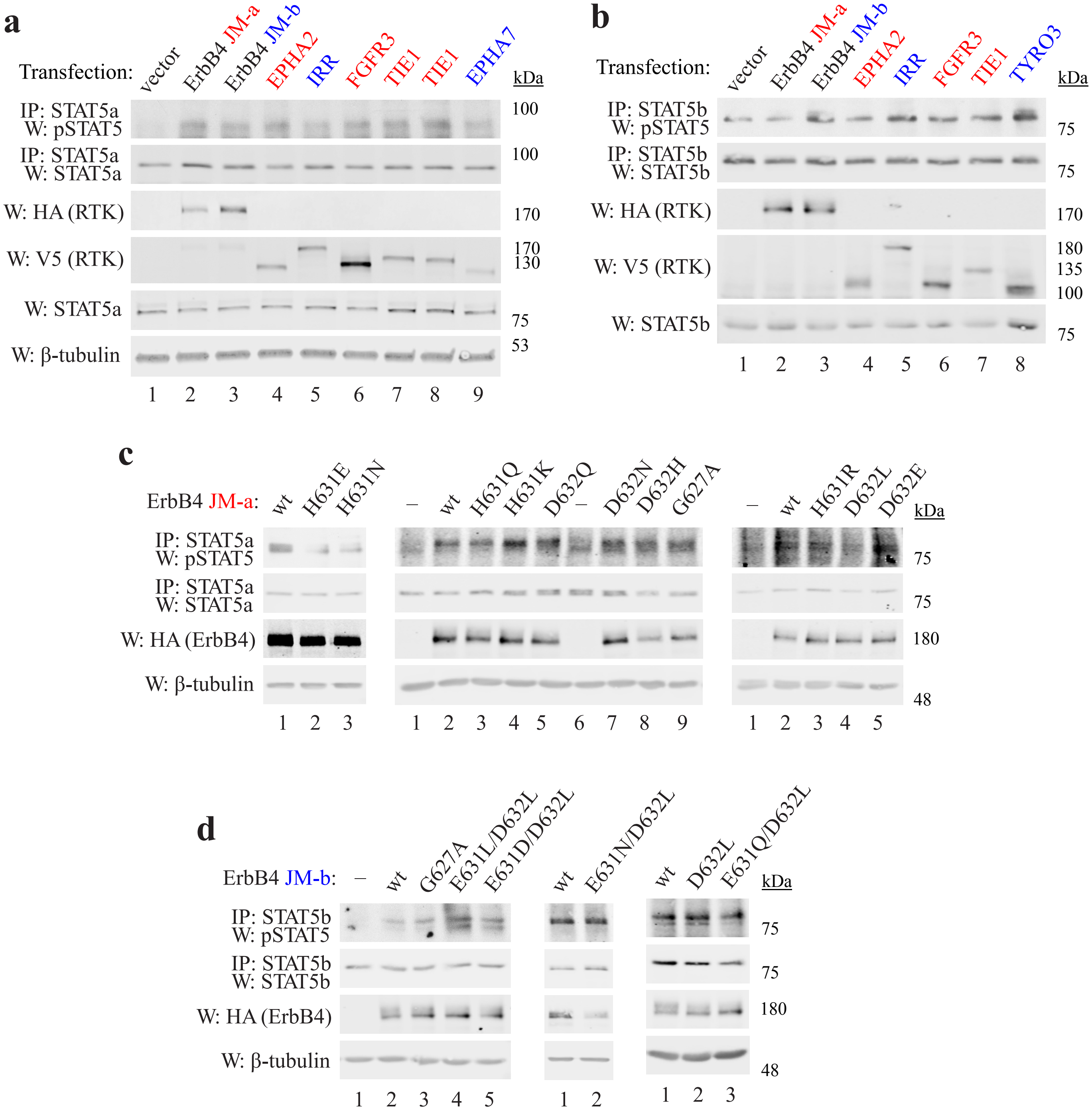

Supplementary Figure 10. Selective STAT5 activation by different RTKs.
a-b: Phosphorylation of STAT5a (a) and STAT5b (b) (on Y694/699) in COS-7 cells expressing ErbB4 JM-a, ErbB4 JM-b, EPHA2, IRR, FGFR3, TIE1, TYRO3 or EPHA7. Cells were treated with 1% FCS. Representative results of n=5 independent experiments.
c-d: Phosphorylation of STAT5a (c) and STAT5b (d) (on Y694/699) in COS-7 cells expressing the indicated wild-type (wt) or mutant ErbB4 constructs. Cells were treated with NRG-1. Representative results of n=3-5 independent experiments.
Source data are provided as a Source Data file.

	ErbB4 JM-a		JM-a-like RTKs	
**Location annotation**	**Enrichment score**	**P-value**	**Enrichment score**	**P-value**
ruffle membrane (GO:0032587)	10.11	0.00354	21.45	0.00056
supramolecular complex (GO:0099080)	2.61	0.00292	25.42	0.0039
subapical part of cell (GO:0120219)	> 100	0.00609	18.06	0.000873
cell cortex region (GO:0099738)	16.76	0.00696	17.6	0.00728
cytoplasmic ribonucleoprotein granule (GO:0036464)	5.31	0.00714	16.34	0.00828
microtubule cytoskeleton (GO:0015630)	2.42	0.0078	16.34	0.00828
respiratory chain complex I (GO:0045271)	13.34	0.0106	8.58	0.00613
MutSbeta complex (GO:0032302)	> 100	0.00912	11.44	0.0154
oxoglutarate dehydrogenase complex (GO:0045252)	65.36	0.0182	3.94	0.0206
mitochondrial inner membrane (GO:0005743)	3.31	0.0179	4.46	0.0324
motile cilium (GO:0031514)	4.02	0.0397	1.9	0.0144
spliceosomal snRNP complex (GO:0097525)	11.27	0.0145	5.87	0.0491

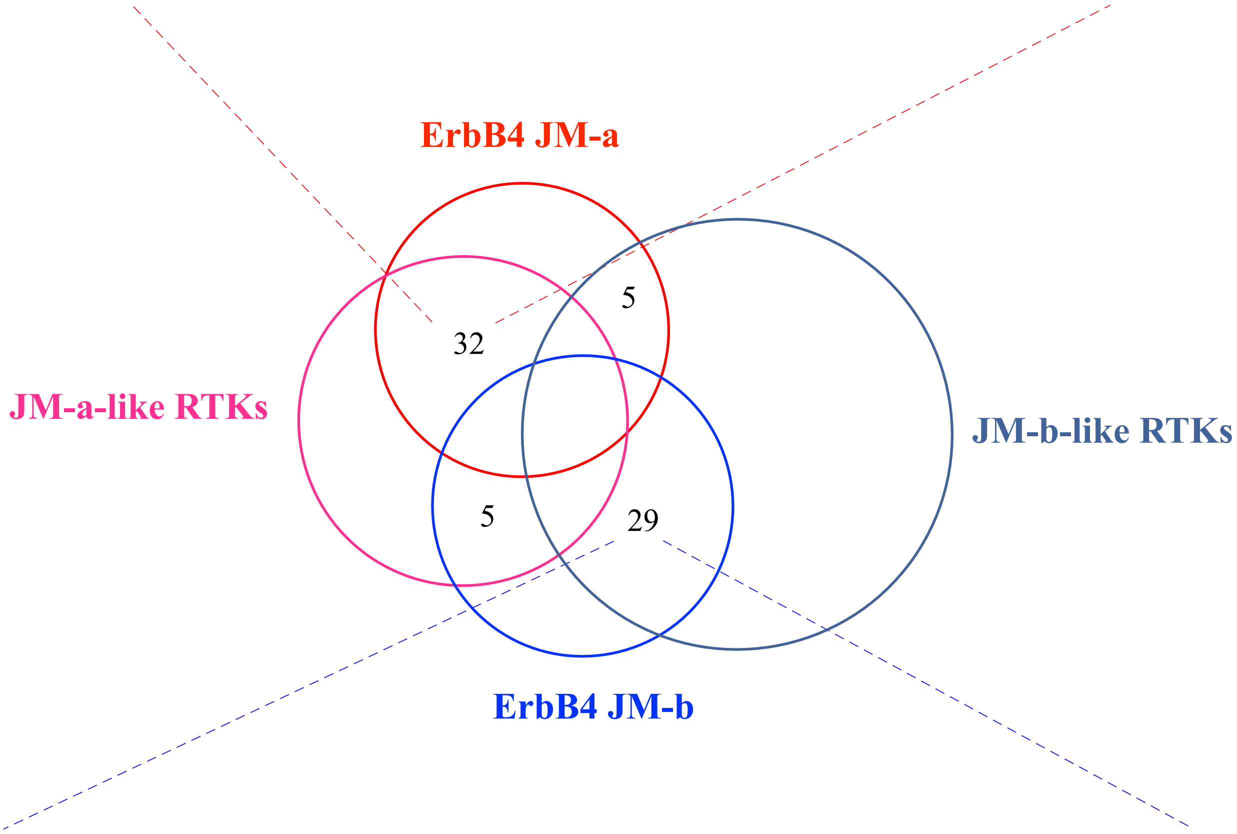

	ErbB4 JM-b		JM-b-like RTKs	
**Location annotation**	**Enrichment score**	**P-value**	**Enrichment score**	**P-value**
endomembrane system (GO:0012505)	1.47	0.0195	6.05	0.00175
RNA polymerase II. holoenzyme (GO:0016591)	7.39	0.00852	10.95	0.0166
ribonucleoprotein complex (GO:1990904)	4.42	3.74E-06	57.51	0.0256
nuclear outer membrane-endoplasmic reticulum membrane network (GO:0042175)	2.01	0.0246	4.87	0.00429
lysosome (GO:0005764)	3.22	0.000662	38.34	0.034
mitochondrial ribosome (GO:0005761)	9.63	0.000929	38.34	0.034
endosome (GO:0005768)	2.73	0.000973	38.34	0.034
membrane protein complex (GO:0098796)	2.41	0.00134	38.34	0.034
intracellular protein-containing complex (GO:0140535)	2.26	0.0262	4.69	0.0118
chromosomal region (GO:0098687)	4.06	0.00188	38.34	0.034
bounding membrane of organelle (GO:0098588)	1.63	0.0428	3.08	0.000406
nuclear envelope (GO:0005635)	2.68	0.0259	4.73	0.028

Supplementary Figure 11. Localization enrichment analysis of the interactomes of ErbB4 JM-a- and JM-b-like RTKs.
The mass spectrometry-derived interactomes of ErbB4 JM-a and ErbB4 JM-b (Supplementary Fig. 2; Supplementary Data 1-4), as well as the interactomes of JM-a- and JM-b-like RTKs (Supplementary Data 6) were subjected to location enrichment analysis with PANTHER Overrepresentation Test (Released 2022-02-02), using GO cellular component annotation dataset (v 10.5281, Released 2022-03-22) and the Fisher's Exact test. The enriched locations of ErbB4 JM-a were found to significantly overlap with locations of the JM-a-like RTKs, and the enriched locations of ErbB4 JM-b to significantly overlap with locations of the JM-b-like RTKs ($P < 0.0001$; χ^2 test). The 12 mostly overlapping location annotations are shown for both JM-a- and JM-b-like receptors. Source data are provided as a Source Data file.

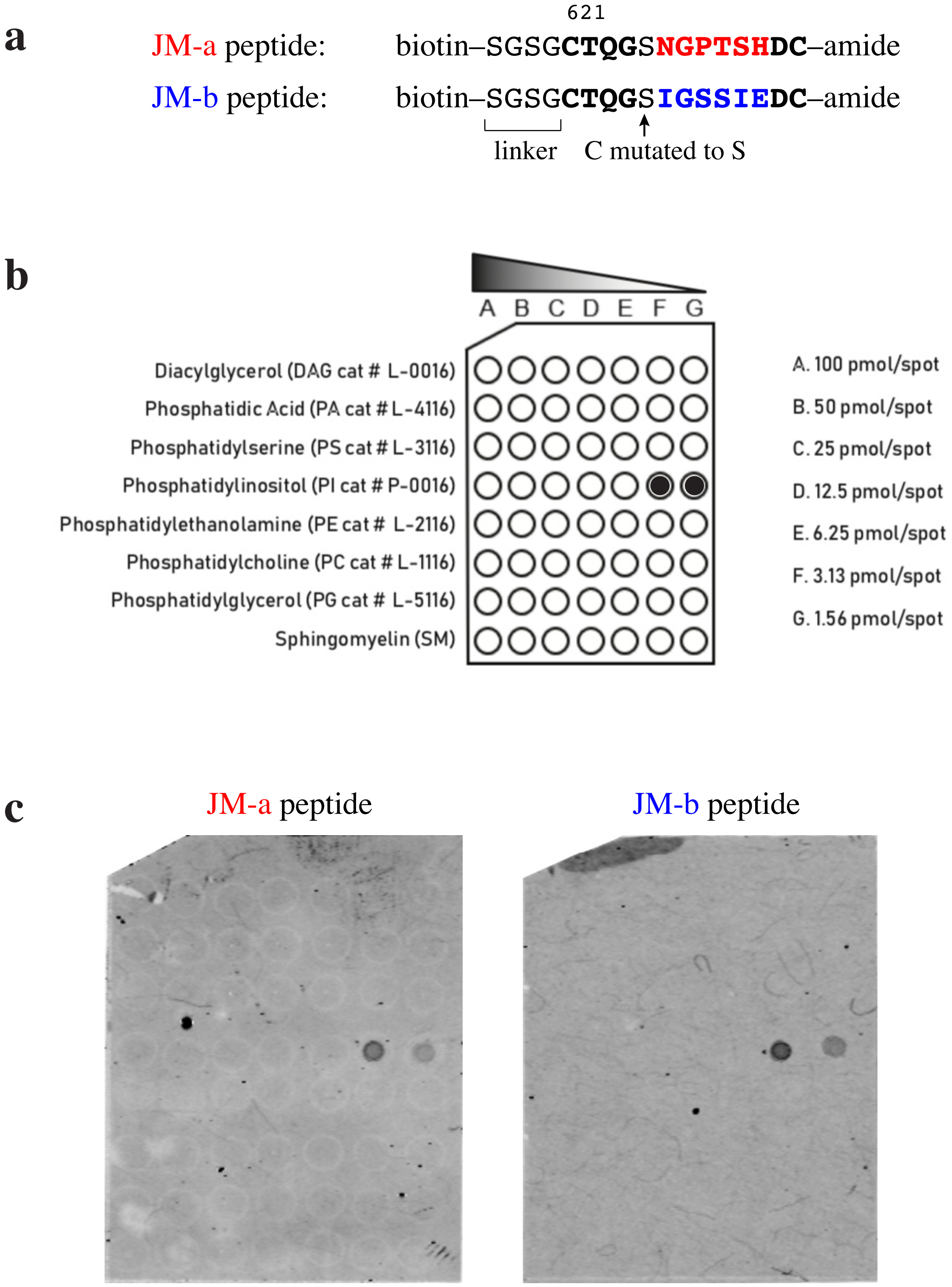

Supplementary Figure 12. Interactions of ErbB4 isoforms with lipids.
a: The indicated biotinylated peptides including the ErbB4 amino acids 621 to 633 were tested for lipid interactions. The cysteine residue 625 was mutated to serine to avoid formation of disulfide bridges other than the natural one between cysteine residues 621 and 633. Isoform-specific residues are indicated with color and ErbB4-derived sequences in bold font.
b-c: Lipid interaction array. Panel b depicts the template by Echelon Bioscience, panel c images of peptide overlay experiments carried out with the peptides indicated in panel a. Phosphatidyl inositol dots interacting with both ErbB4 isoforms are indicated by filled circles in panel b. Representative blots of n=2 independent experiments.

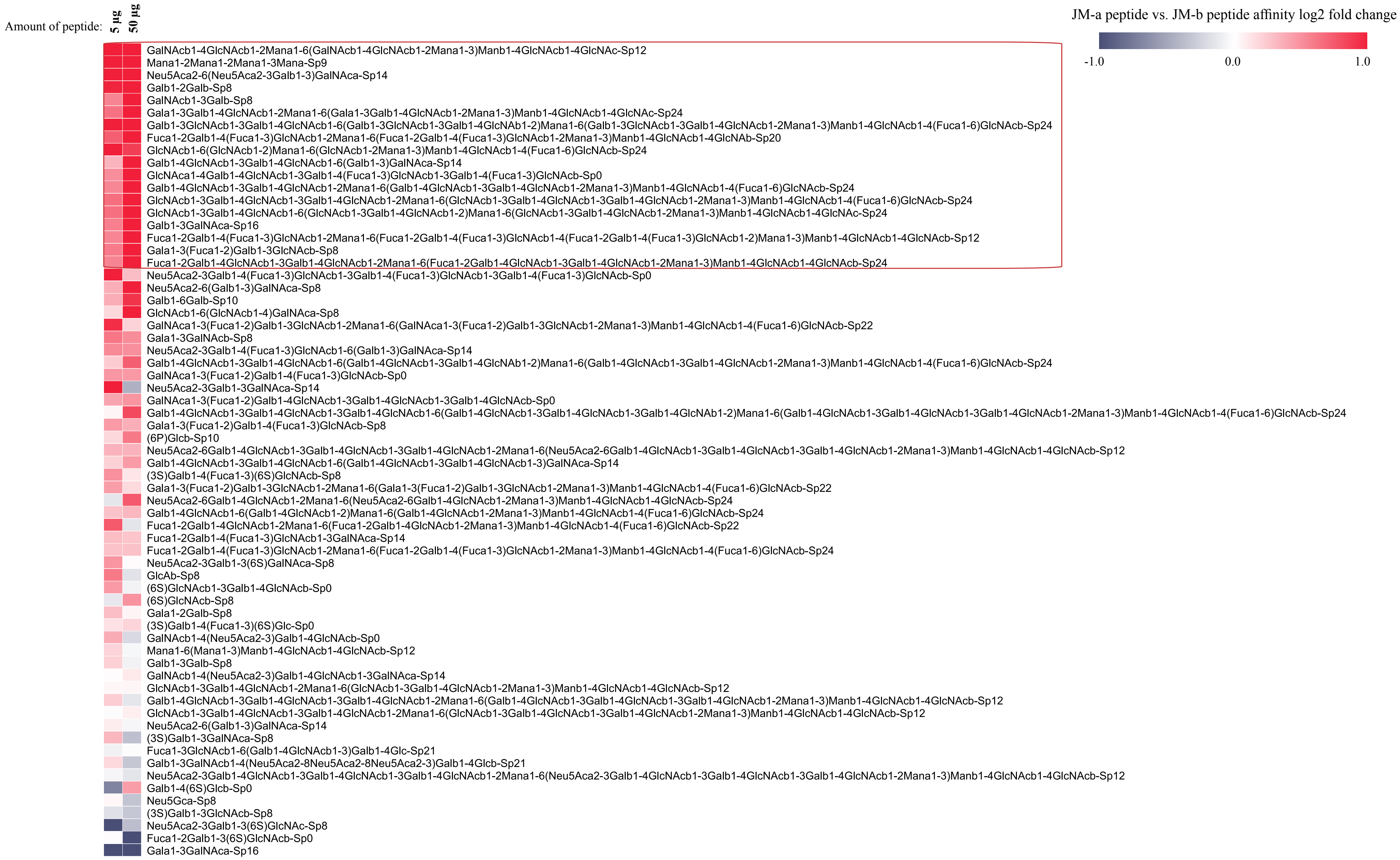

Supplementary Figure 13. Interactions of ErbB4 isoforms with glycans.
Peptides representing the amino acid residues 621 to 633 of ErbB4 JM-a and JM-b (Supplementary Fig. 12a) were analyzed for interactions with glycans using a mammalian glycan array version 5.2 from CFG. The results are shown as a heatmap demonstrating the relative affinity of the interaction of the two peptides with the indicated glycan species. The 18 oligosaccharides with the greatest relative affinity to JM-a-specific peptide as compared to JM-b peptide (also shown in Fig. 6a) are bounded by a red box. Source data are provided as a Source Data file.

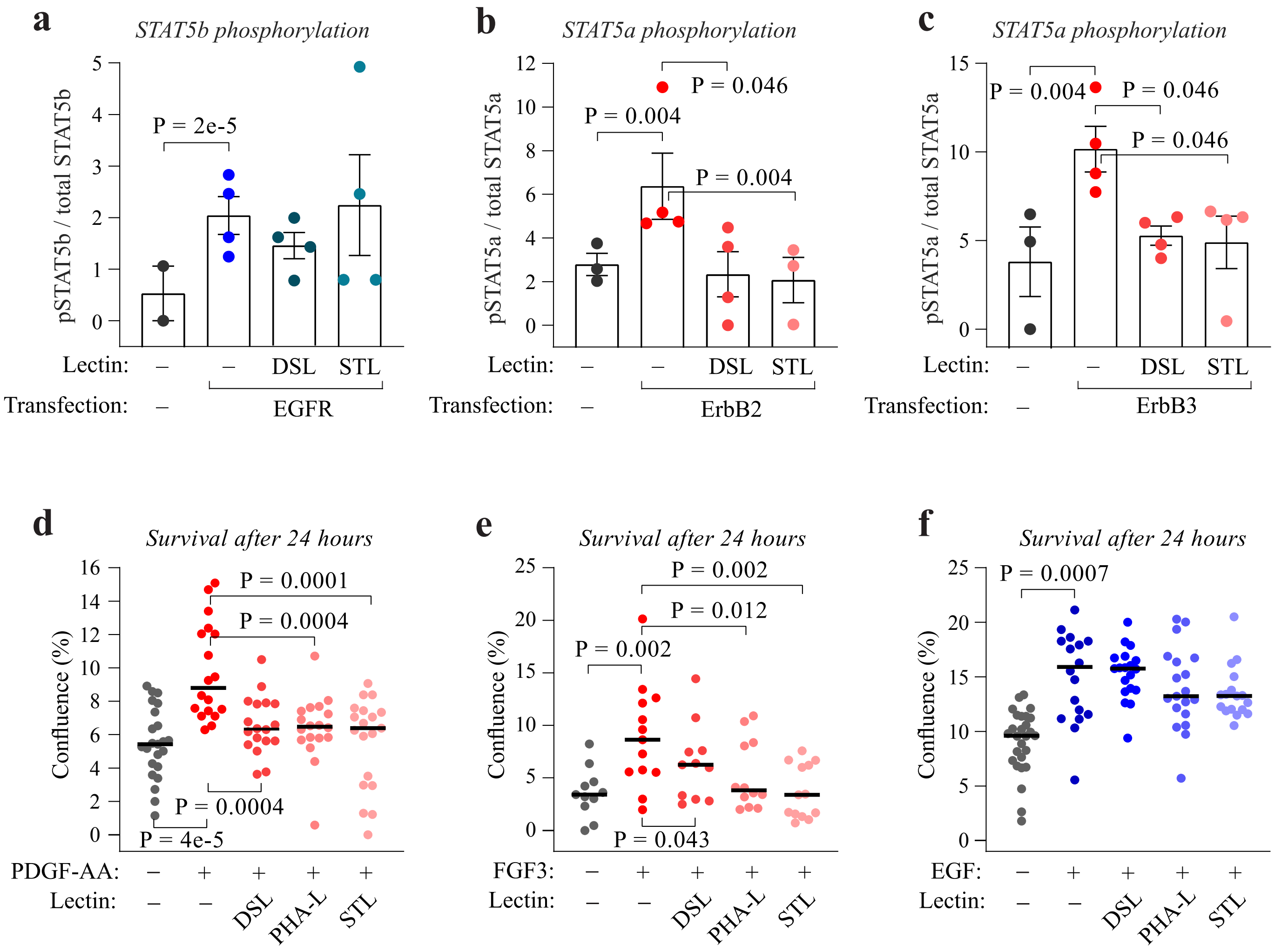

Supplementary Figure 14. Lectin-sensitivity of selective STAT5 activation and cell survival stimulated by RTKs.

a-c: The indicated lectins were analyzed for their potential to inhibit ErbB-stimulated STAT5 activation. Panels depict densitometric quantification of data from Western analyses of STAT5b (a) or STAT5a (b and c) phosphorylation (on Y694/699) in COS-7 cells overexpressing EGFR (a), ErbB2 (b) or ErbB3 (c). Cells were treated with NRG-1. Mean ± SEM. n=3-4 independent experiments. Mack-Skillings two-way ANOVA.

d-f: Growth of serum-starved HC11 cells cultured in the presence of the indicated lectins and either PDGF-AA (d), FGF3 (e) or EGF (f). Cell confluence was measured using live cell imaging with IncuCyte. n=16-33 wells examined over 3 independent experiments. Brown-Forsythe ANOVA. Benjamini, Krieger and Yekutieli adjusted P-values.
Source data are provided as a Source Data file.

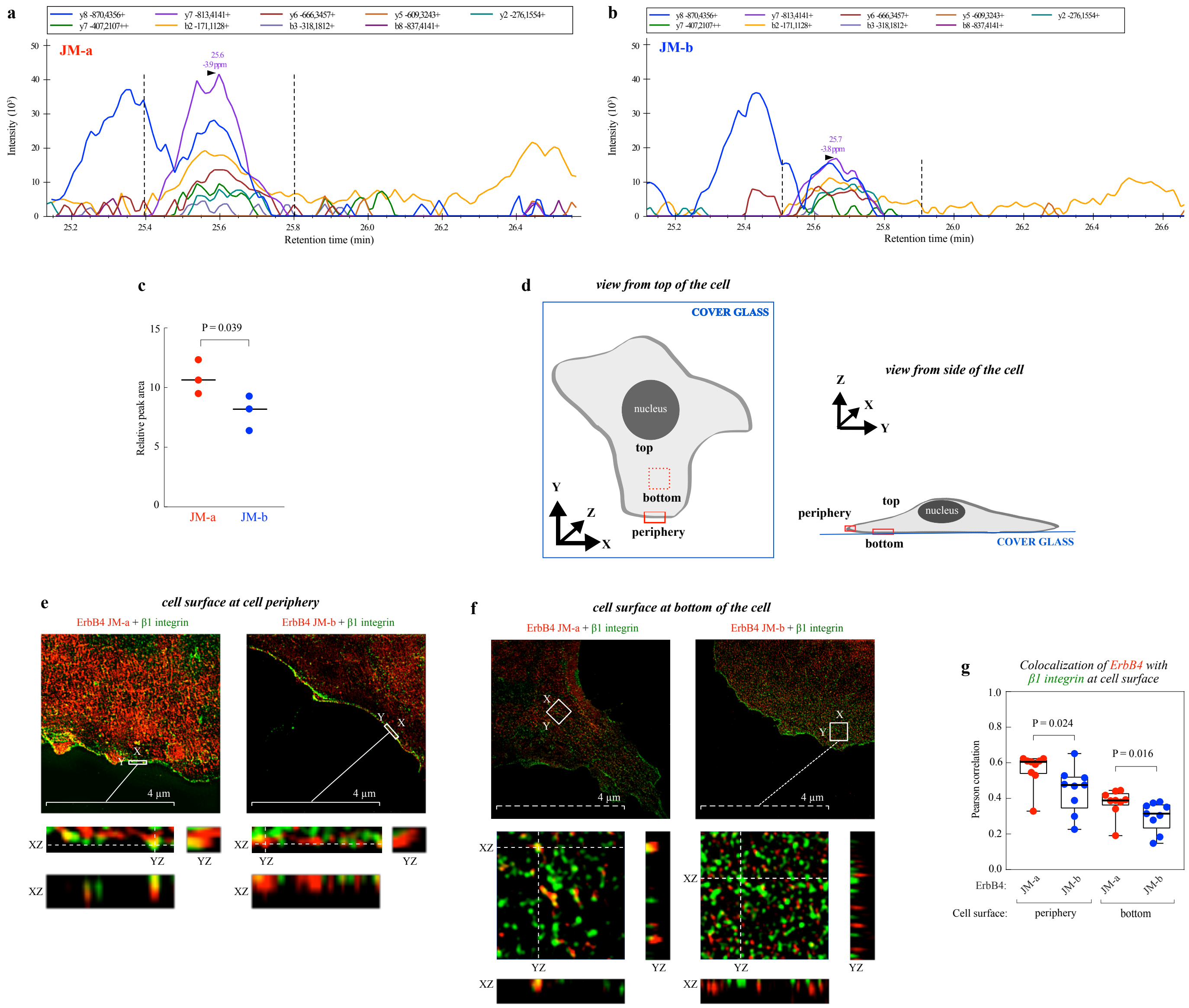

Supplementary Figure 15. Association of ErbB4 JM isoforms with β1 integrin at different compartments of the cell surface.

a-b: Representative fragment ion peaks of the unique β1 integrin peptide IGFGSFVEK in anti-ErbB4 immunoprecipitates of MDA-MB-468 cells expressing ErbB4 JM-a (a) or ErbB4 JM-b (b). The peptide was detected with parallel reaction monitoring (PRM) technique by mass spectrometry and analyzed with Skyline. Included transitions are indicated above the peak curves. The vertical dashed lines indicate the boundaries of the quantification. ppm, parts per million.

c: Sums of the areas of the fragment ion peaks of the unique β1 integrin peptide IGFGSFVEK precipitating with ErbB4 JM-a or JM-b in n=3 independent PRM experiments such as shown in panels a and b. Two-tailed T-test.

d: Schematic representation of the imaging strategy.

e-g: SIM super-resolution immunofluorescence analysis of colocalization of endogenously expressed β1 integrin (green) and ectopically expressed ErbB4 isoforms (red) in COS-7 cells. The imaging was focused at the cell surfaces representing peripheral cell surfaces at the side (e), and bottom surfaces facing the cover glass at the culture plate (f). The white boxes highlight the regions of interest in the x-y plane that are magnified below. The white dashed lines correspond to the position of the x-z (below the magnified view) and y-z (right of the magnified view) projections in the x-y image. Panel g depicts quantification of co-localization of anti-ErbB4- and anti-β1 integrin epitope signals at the different cell surfaces in the x-y plane where each dot represents the correlation of the signals in one cell. n=9 cells examined over 2 independent experiments. Two-tailed Whitney U test. In the boxplots the line represents the median, the box the interquartile range and whiskers the whole range of values.

Source data are provided as a Source Data file.
