# Supplementary material for: An extracellular receptor tyrosine kinase motif orchestrating intracellular STAT activation
Source: Nat Commun. 2022 Nov 14;13:6953. doi: 10.1038/s41467-022-34539-4 (PMC9663514; doi:10.1038/s41467-022-34539-4)
Supplement: Supplementary file 3 — Description of Additional Supplementary Files [file 41467_2022_34539_MOESM3_ESM.pdf]

Description of Additional Supplementary Files

File Name: Supplementary Data 1

Description: The mass spectrometry derived interactome of ErbB4 JM-a and JM-b in MDA-MB-468 cells, experiment 1.

File Name: Supplementary Data 2

Description: The mass spectrometry derived interactome of ErbB4 JM-a and JM-b in MDA-MB-468 cells, experiment 2.

File Name: Supplementary Data 3

Description: The mass spectrometry derived interactome of ErbB4 JM-a and JM-b in MDA-MB-468 cells, experiment 3.

File Name: Supplementary Data 4

Description: The mass spectrometry derived interactome of ErbB4 JM-a and JM-b in MDA-MB-468 cells, experiment 4.

File Name: Supplementary Data 5

Description: Performance of all examined sequence models in categorizing JM-a and JM-b-like RTKs.

File Name: Supplementary Data 6

Description: The MS-AP and BIO-ID derived interactomes of JM-a and JM-b-like RTKs.

File Name: Supplementary Data 7

Description: Subcellular location enrichment of the interactomes of ErbB4 JM-a and JM-b and JM-a and JM-b- like RTKs.

File Name: Supplementary Data 8

Description: The affinity of ErbB4 JM-a and JM-b peptides on the CFG mammalian glycan array.

File Name: Supplementary Data 9

Description: The mass-spectrometry-derived affinity enrichment results from the PHA-L lectin pull-down in COS-7, HC11 and MCF-7 cells.

File Name: Supplementary Data 10

Description: Resources table.
