# Supplementary material for: An extracellular receptor tyrosine kinase motif orchestrating intracellular STAT activation
Source: Nat Commun. 2022 Nov 14;13:6953. doi: 10.1038/s41467-022-34539-4 (PMC9663514; doi:10.1038/s41467-022-34539-4)
Supplement: Supplementary file 13 — Supplementary Dataset 10 [file 41467_2022_34539_MOESM13_ESM.docx]

**RESOURCES TABLE**

| **REAGENT or RESOURCE** | **SOURCE** | **IDENTIFIER** |
| --- | --- | --- |
| **Antibodies & Lectins*** | | |
| Mouse monoclonal anti-β-actin (AC-74) (W) | Sigma | A5441 |
| Goat polyclonal anti-Actin (I-19) (W) | Santa Cruz | sc-1616 |
| Mouse monoclonal anti-β1-integrin (4B7R) (IF) | Santa Cruz | sc-9970 |
| Mouse monoclonal anti-β1-integrin (K-20) (PLA) | Santa Cruz | sc-18887 |
| Rabbit monoclonal anti-β1-integrin (EP1041Y) (W) | Abcam | ab52971 |
| Rat monoclonal anti-β1-integrin (Mab 13) (IP) | BD Pharmingen | 552828 |
| Rabbit monoclonal anti-β4-integrin (D8P6C) (W) | Cell Signaling | #14803 |
| Rabbit polyclonal anti-β-Tubulin (H-235) (W) | Santa Cruz | sc-9104 |
| Mouse monoclonal anti-β-Tubulin (SAP.4G5) (W) | Sigma | T7816 |
| Rabbit polyclonal EGF Receptor Antibody (W) | Cell Signaling | #2232 |
| Rabbit monoclonal Phospho-EGF Receptor (Tyr1068) (D7A5) (W) | Cell Signaling | #3777 |
| Rabbit polyclonal ErbB2 Antibody (C-18) | Santa Cruz | sc-284 |
| Rabbit monoclonal Phospho-HER2/ErbB2 (Tyr1248) Antibody (W) | Cell Signaling | #2247 |
| Rabbit monoclonal anti-ErbB4 (E200) (IF,IP, W, PLA) | Abcam | ab32375 |
| Mouse monoclonal anti-ErbB4 (HFR-1) (IF, IP) | Abcam | ab19391 |
| Rabbit monoclonal anti- Phospho-HER4/ErbB4 (Tyr1284) (21A9) (W) | Cell Signaling | #4757 |
| Rabbit polyclonal anti-GFP (IP,W) | Abcam | ab6556 |
| Mouse monoclonal anti-HA-Tag (6E2) (W, IP, PLA) | Cell Signaling | #2367 |
| Mouse monoclonal anti-HA (HA-7) (IF) | Sigma | H3663 |
| Rabbit monoclonal anti-JAK2 (DE12) (W) | Cell Signaling | #3230 |
| Rabbit polyclonal anti-JAK2 (HR-758) (W) | Santa Cruz | sc-278 |
| Rabbit monoclonal anti-Lamin B1 (D4Q4Z) (W) | Cell Signaling | #12586 |
| Goat polyclonal anti-Lamin B (M-20) (W) | Santa Cruz | sc-6217 |
| Rabbit polyclonal anti-Myc-Tag (IF) | Cell Signaling | #2272 |
| Rabbit monoclonal p44/42 MAPK (Erk1/2) (W) | Cell Signaling | # 9102 |
| Rabbit polyclonal P-p44/42 MAPK (T202/Y204) | Cell Signaling | # 9101 |
| Rabbit polyclonal anti-PDGFRα antibody (W) | Millipore | 07-276 |
| Rabbit polyclonal anti-PEPP3 (W) | Abcam | ab173483 |
| Mouse monoclonal anti- POL II (8WG16) (W) | Santa Cruz | sc-56767 |
| Rabbit monoclonal anti-Sodium Potassium ATPase (EP1845Y) (IF, W) | Abcam | ab76020 |
| Mouse monoclonal Stat3 (124H6) (W) | Cell Signaling | #9139 |
| Rabbit monoclonal Phospho-Stat3 (Tyr705) (D3A7) XP® (W) | Cell Signaling | #9145 |
| Mouse monoclonal Phospho-Stat5 (Tyr694) (14H2) (W) | Cell Signaling | #9356 |
| Rabbit polyclonal anti-Phospho-STAT5 (Tyr694) (W) | Cell Signaling | #9351 |
| Mouse monoclonal anti-STAT5a (C-6)(IP,W) | Santa Cruz | sc-271542 |
| Rabbit polyclonal anti-STAT5a (L-20) (IF, IP, W) | Santa Cruz | sc-1081 |
| Rabbit polyclonal anti-STAT5a (Ab 780) (IP, W) | Sigma | SAB4300330 |
| Rabbit polyclonal anti-STAT5a Prestige Antibodies (IP, PLA) | Sigma | HPA027873 |
| Rabbit polyclonal anti-STAT5 (C-17) (recognizes STAT5b) (IF, IP, W) | Santa Cruz | sc-835 |
| Rabbit oligoclonal anti- STAT5b (13HCLC) (IP) | Thermo Fisher Scientific | 710139 |
| Rabbit polyclonal anti-STAT5b (IF, IP, W) | R&D | AF1584 |
| Mouse monoclonal anti-STAT5B antibody (clone 2D1) (PLA) | Sigma | SAB1412214 |
| Rabbit polyclonal anti-TYK2 (W) | Cell Signaling | #9312 |
| Mouse monoclonal anti-p-Tyr (PY99 )HRP (W) | Santa Cruz | sc-7020 HRP |
| IRDye 680RD Donkey anti-Goat IgG (H + L) (W) | LI-COR | 925-68074 |
| IRDye 800CW Donkey anti-Goat IgG (H + L) (W) | LI-COR | 925-32214 |
| rabbit anti-goat IgG-HRP (W) | Santa Cruz | sc-2768 |
| Goat anti-Mouse IgG (H+L) Highly Cross-Adsorbed Secondary Antibody, Alexa Fluor 488 (IF) | Invitrogen | A-11029 |
| Goat anti-Mouse IgG (H+L) Highly Cross-Adsorbed Secondary Antibody, Alexa Fluor 555 (IF) | Invitrogen | A-21424 |
| IRDye 680RD Donkey anti-Mouse IgG (H + L) (W) | LI-COR | 925-68072 |
| IRDye 800CW Donkey anti-Mouse IgG (H + L) (W) | LI-COR | 925-32212 |
| goat anti-mouse IgG-HRP (W) | Santa Cruz | sc-2005 |
| Goat anti-Rabbit IgG (H+L) Highly Cross-Adsorbed Secondary Antibody, Alexa Fluor 488 (IF) | Invitrogen | A-11034 |
| Goat anti-Rabbit IgG (H+L) Highly Cross-Adsorbed Secondary Antibody, Alexa Fluor 555 (IF) | Invitrogen | A-21429 |
| IRDye 680RD Donkey anti-Rabbit IgG (H + L) (W) | LI-COR | 926-68073 |
| IRDye 800CW Donkey anti-Rabbit IgG (H + L) (W) | LI-COR | 925-32213 |
| goat anti-rabbit IgG-HRP (W) | Santa Cruz | sc-2004 |
| Goat anti-Rabbit IgG (H+L) Cross-Adsorbed Secondary Antibody, HRP (W) | Invitrogen | A16104 |
| Goat anti-rabbit IgG Abberior STAR 635 (IF) | Abberior | 2-0012-002-7 |
| Abberior STAR 580, goat anti-mouse IgG (IF) | Abberior | 2-0002-005-1 |
| Biotinylated Aleuria Aurantia Lectin | Vector labs | B-1395-1 |
| Biotinylated Datura Stramonium Lectin | Vector labs | B-1185-2 |
| Biotinylated Solanum Tuberosum (Potato) Lectin | Vector labs | B-1165-2 |
| Biotinylated Erythrina Cristagalli Lectin | Vector labs | B-1145-5 |
| Biotinylated Maackia Amurensis I Lectin | Vector labs | B-1315-2 |
| Biotinylated Galanthus Nivalis Lectin | Vector labs | B-1245-2 |
| Biotinylated Narcissus Pseudonarcissus Lectin | Vector labs | B-1375-2 |
| Biotinylated Griffonia Simplicifolia I Lectin | Vector labs | B-1105-2 |
| Biotinylated Ricinus Communis I (120) Agglutinin | Vector labs | B-1085-5 |
| Biotinylated Lotus Lectin | Vector labs | B-1325-2 |
| Biotinylated Ulex Europaeus I Agglutinin | Vector labs | B-1065-2 |
| Biotinylated Maackia Amurensis Lectin Ii | Vector labs | B-1265-1 |
| Biotinylated Phaseolus Vulgaris Leucoagglutinin, PHA-L | Vector labs | B-1115-2 |
| Biotinylated Phaseolus Vulgaris Erythroagglutinin | Vector labs | B-1125-2 |
| Biotinylated Sambucus Nigra Lectin | Vector labs | B-1305-2 |
| **Chemicals and reagents** |  |  |
| BS_3_ (bis(sulfosuccinimidyl)suberate) | Thermo Fisher Scientific | 21580 |
| DAPI | Sigma-Aldrich | D9542 |
| DMSO, Dimethyl Sulfoxide, Fisher BioReagents | Thermo Fisher Scientific | BP231-100 |
| DSP (dithiobis(succinimidyl propionate)), Lomant's Reagent | Thermo Fisher Scientific | 22585 |
| EGF human, recombinant | Sigma | E9644 |
| Geneticin Selective Antibiotic (G418 Sulfate) (50 mg/mL) | Gibco | 10131-019 |
| FastAP Thermosensitive Alkaline Phosphatase (1 U/µL) | Thermo Fisher Scientific | EF0654 |
| Recombinant Human FGF-3 Protein | R&D Systems | 1206-F3-025 |
| PureProteome Protein G Magnetic Bead System | Millipore | LSKMAGG10 |
| Protein G-sepharose 4 Fast Flow | GE Healthcare | 17-0618-01 |
| Gateway LR Clonase II Enzyme mix | Thermo Fisher Scientific | 11791100 |
| GSI-IX | Calbiochem | 565784 |
| HBSS | Thermo Fisher Scientific | 88284 |
| JAK kinase inhibitor I | Calbiochem | 420099 |
| JAK2 kinase inhibitor V | Calbiochem | 420141 |
| KAPA HiFi PCR Kit | Kapa Biosystems | KK2101 |
| NEBuilder HiFi DNA Assembly Master Mix | New England Biolabs | E2621 |
| Recombinant Human NRG1-beta 1 | R&D Systems | 396-HB |
| Mowiol 4-88 | Sigma | 475904 |
| Recombinant Human PDGF-AA Protein, CF | R&D Systems | 221-AA-010 |
| Phusion High-Fidelity DNA Polymerase (2 U/µL) | Thermo Fisher Scientific | F530S |
| Pierce Protease and Phosphatase Inhibitor Mini Tablets | Thermo Fisher Scientific | 88668 |
| The NaveniFlex MR kit is in situ proximity ligation assay kit | Navinci | NaveniFlex MR |
| Polybrene | Sigma | 28728-55-4 |
| Puromycin Dihydrochloride | Gibco | A1113803 |
| Restriction enzyme, BbSI Fast Digest | Thermo Fisher Scientific | FD1014 |
| Restriction enzyme, Bsp1407I Fast Digest | Thermo Fisher Scientific | FD0933 |
| Restriction enzyme, EcoRV Fast Digest | Thermo Fisher Scientific | FD0303 |
| Restriction enzyme, KpnI Fast Digest | Thermo Fisher Scientific | FD0524 |
| Restriction enzyme, XcmI | New England Biolabs | R0533 |
| ReproSil-Pur 5 µm 200 Å C18-AQ beads | Dr. Maisch | r25.aq. |
| SensiFAST cDNA Synthesis Kit | Bioline | BIO-65054 |
| Streptavidin Sepharose High Performance | GE Healthcare | GE17-5113-01 |
| TAPI-0 | Calbiochem | 579050 |
| T4 DNA Ligase (5 U/µL) | Thermo Fisher Scientific | EL0011 |
| Trisure | Bioline | BIO-38033 |
| **Oligonucleotides** |  |  |
| pBABE-puro ErbB4 JM-a CYT-2-HA, FWRD:  TATGCTAGAAGGAAGA GCATC | Eurofins Genomics |  |
| pBABE-puro ErbB4 JM-a CYT-2-HA, REV:  AACAGCAAATGTCAGACC | Eurofins Genomics |  |
| pcDNA3.1 ErbB4 JM-a G627A CYT-2 –HA, GENEFragment:  GCCATGGACCGGGTCCTGACAACTGTACAAAGTGCTCTCATTTTAAAGATGGCCCAAACTGTGTGGAAAAATGTCCAGATGGCTTACAGGGGGCAAACAGTTTCATTTTCAAGTATGCTGATCCAGATCGGGAGTGCCACCCATGCCATCCAAACTGCACCCAAGGGTGTAACGCTCCCACTAGTCATGACTGCATTTACTACCCATGGACGGGCCATTCCACTTTACCACAACATGCTAGAACTCCCCTGATTGCAGCTGGAGTAATTGGTGGGCTCTTCATTCTGGTCATTGTGGGTCTGACATTTGCTGTTTATGTTAGAAGGAAGAGCATCAAAAAGAAAAGAGCCTTGAGAAGATTCTTGGAAACAGAGTTGGTGGAACCATTAACTCCCAGTGGCACAGCACCCAATCAAGCTCAACTTCGTATTTTGAAAGAAACTGAGCTGAAGAGGGTAAAAGTCCTTGGCTCAGGTGCTTTTGGAACGGTTTATAAAGGTATTTGGGTACCTGAAGGAGAAACTGTGAAGATTCCTG | Gene Universal |  |
| pcDNA3.1 ErbB4 JM-a H631E CYT-2 –HA, FWRD:  GTAACGGTCCCACTAGTGAAGACTGCATTTAC | Eurofins Genomics |  |
| pcDNA3.1 ErbB4 JM-a H631E CYT-2 –HA, REV:  CATGGGTAGTAAATGCAGTCTTCACTAGTGGG | Eurofins Genomics |  |
| pcDNA3.1 ErbB4 JM-a H631K CYT-2 –HA, GENEFragment:  GCCATGGACCGGGTCCTGACAACTGTACAAAGTGCTCTCATTTTAAAGATGGCCCAAACTGTGTGGAAAAATGTCCAGATGGCTTACAGGGGGCAAACAGTTTCATTTTCAAGTATGCTGATCCAGATCGGGAGTGCCACCCATGCCATCCAAACTGCACCCAAGGGTGTAACGGTCCCACTAGTAAGGACTGCATTTACTACCCATGGACGGGCCATTCCACTTTACCACAACATGCTAGAACTCCCCTGATTGCAGCTGGAGTAATTGGTGGGCTCTTCATTCTGGTCATTGTGGGTCTGACATTTGCTGTTTATGTTAGAAGGAAGAGCATCAAAAAGAAAAGAGCCTTGAGAAGATTCTTGGAAACAGAGTTGGTGGAACCATTAACTCCCAGTGGCACAGCACCCAATCAAGCTCAACTTCGTATTTTGAAAGAAACTGAGCTGAAGAGGGTAAAAGTCCTTGGCTCAGGTGCTTTTGGAACGGTTTATAAAGGTATTTGGGTACCTGAAGGAGAAACTGTGAAGATTCCTG | Gene Universal |  |
| pcDNA3.1 ErbB4 JM-a H631N CYT-2 –HA, FWRD:  GTAACGGTCCCACTAGTAACGACTGCATTTAC | Eurofins Genomics |  |
| pcDNA3.1 ErbB4 JM-a H631N CYT-2 –HA, REV:  CATGGGTAGTAAATGCAGTCATTACTAGTGGG | Eurofins Genomics |  |
| pcDNA3.1 ErbB4 JM-a H631R CYT-2 –HA, GENEFragment:  GCCATGGACCGGGTCCTGACAACTGTACAAAGTGCTCTCATTTTAAAGATGGCCCAAACTGTGTGGAAAAATGTCCAGATGGCTTACAGGGGGCAAACAGTTTCATTTTCAAGTATGCTGATCCAGATCGGGAGTGCCACCCATGCCATCCAAACTGCACCCAAGGGTGTAACGGTCCCACTAGTCGTGACTGCATTTACTACCCATGGACGGGCCATTCCACTTTACCACAACATGCTAGAACTCCCCTGATTGCAGCTGGAGTAATTGGTGGGCTCTTCATTCTGGTCATTGTGGGTCTGACATTTGCTGTTTATGTTAGAAGGAAGAGCATCAAAAAGAAAAGAGCCTTGAGAAGATTCTTGGAAACAGAGTTGGTGGAACCATTAACTCCCAGTGGCACAGCACCCAATCAAGCTCAACTTCGTATTTTGAAAGAAACTGAGCTGAAGAGGGTAAAAGTCCTTGGCTCAGGTGCTTTTGGAACGGTTTATAAAGGTATTTGGGTACCTGAAGGAGAAACTGTGAAGATTCCTG | Gene Universal |  |
| pcDNA3.1 ErbB4 JM-a H631Q CYT-2 –HA, GENEFragment:  GCCATGGACCGGGTCCTGACAACTGTACAAAGTGCTCTCATTTTAAAGATGGCCCAAACTGTGTGGAAAAATGTCCAGATGGCTTACAGGGGGCAAACAGTTTCATTTTCAAGTATGCTGATCCAGATCGGGAGTGCCACCCATGCCATCCAAACTGCACCCAAGGGTGTAACGGTCCCACTAGTCAAGACTGCATTTACTACCCATGGACGGGCCATTCCACTTTACCACAACATGCTAGAACTCCCCTGATTGCAGCTGGAGTAATTGGTGGGCTCTTCATTCTGGTCATTGTGGGTCTGACATTTGCTGTTTATGTTAGAAGGAAGAGCATCAAAAAGAAAAGAGCCTTGAGAAGATTCTTGGAAACAGAGTTGGTGGAACCATTAACTCCCAGTGGCACAGCACCCAATCAAGCTCAACTTCGTATTTTGAAAGAAACTGAGCTGAAGAGGGTAAAAGTCCTTGGCTCAGGTGCTTTTGGAACGGTTTATAAAGGTATTTGGGTACCTGAAGGAGAAACTGTGAAGATTCCTG | Gene Universal |  |
| pcDNA3.1 ErbB4 JM-a D632H CYT-2 –HA, GENEFragment:  GCCATGGACCGGGTCCTGACAACTGTACAAAGTGCTCTCATTTTAAAGATGGCCCAAACTGTGTGGAAAAATGTCCAGATGGCTTACAGGGGGCAAACAGTTTCATTTTCAAGTATGCTGATCCAGATCGGGAGTGCCACCCATGCCATCCAAACTGCACCCAAGGGTGTAACGGTCCCACTAGTCATCACTGCATTTACTACCCATGGACGGGCCATTCCACTTTACCACAACATGCTAGAACTCCCCTGATTGCAGCTGGAGTAATTGGTGGGCTCTTCATTCTGGTCATTGTGGGTCTGACATTTGCTGTTTATGTTAGAAGGAAGAGCATCAAAAAGAAAAGAGCCTTGAGAAGATTCTTGGAAACAGAGTTGGTGGAACCATTAACTCCCAGTGGCACAGCACCCAATCAAGCTCAACTTCGTATTTTGAAAGAAACTGAGCTGAAGAGGGTAAAAGTCCTTGGCTCAGGTGCTTTTGGAACGGTTTATAAAGGTATTTGGGTACCTGAAGGAGAAACTGTGAAGATTCCTG | Gene Universal |  |
| pcDNA3.1 ErbB4 JM-a D632L CYT-2 –HA, GENEFragment:  GCCATGGACCGGGTCCTGACAACTGTACAAAGTGCTCTCATTTTAAAGATGGCCCAAACTGTGTGGAAAAATGTCCAGATGGCTTACAGGGGGCAAACAGTTTCATTTTCAAGTATGCTGATCCAGATCGGGAGTGCCACCCATGCCATCCAAACTGCACCCAAGGGTGTAACGGTCCCACTAGTCATCTCTGCATTTACTACCCATGGACGGGCCATTCCACTTTACCACAACATGCTAGAACTCCCCTGATTGCAGCTGGAGTAATTGGTGGGCTCTTCATTCTGGTCATTGTGGGTCTGACATTTGCTGTTTATGTTAGAAGGAAGAGCATCAAAAAGAAAAGAGCCTTGAGAAGATTCTTGGAAACAGAGTTGGTGGAACCATTAACTCCCAGTGGCACAGCACCCAATCAAGCTCAACTTCGTATTTTGAAAGAAACTGAGCTGAAGAGGGTAAAAGTCCTTGGCTCAGGTGCTTTTGGAACGGTTTATAAAGGTATTTGGGTACCTGAAGGAGAAACTGTGAAGATTCCTG | Gene Universal |  |
| pcDNA3.1 ErbB4 JM-a D632N CYT-2 –HA, GENEFragment:  GCCATGGACCGGGTCCTGACAACTGTACAAAGTGCTCTCATTTTAAAGATGGCCCAAACTGTGTGGAAAAATGTCCAGATGGCTTACAGGGGGCAAACAGTTTCATTTTCAAGTATGCTGATCCAGATCGGGAGTGCCACCCATGCCATCCAAACTGCACCCAAGGGTGTAACGGTCCCACTAGTCATAACTGCATTTACTACCCATGGACGGGCCATTCCACTTTACCACAACATGCTAGAACTCCCCTGATTGCAGCTGGAGTAATTGGTGGGCTCTTCATTCTGGTCATTGTGGGTCTGACATTTGCTGTTTATGTTAGAAGGAAGAGCATCAAAAAGAAAAGAGCCTTGAGAAGATTCTTGGAAACAGAGTTGGTGGAACCATTAACTCCCAGTGGCACAGCACCCAATCAAGCTCAACTTCGTATTTTGAAAGAAACTGAGCTGAAGAGGGTAAAAGTCCTTGGCTCAGGTGCTTTTGGAACGGTTTATAAAGGTATTTGGGTACCTGAAGGAGAAACTGTGAAGATTCCTG | Gene Universal |  |
| pcDNA3.1 ErbB4 JM-a D632Q CYT-2 –HA, GENEFragment:  GCCATGGACCGGGTCCTGACAACTGTACAAAGTGCTCTCATTTTAAAGATGGCCCAAACTGTGTGGAAAAATGTCCAGATGGCTTACAGGGGGCAAACAGTTTCATTTTCAAGTATGCTGATCCAGATCGGGAGTGCCACCCATGCCATCCAAACTGCACCCAAGGGTGTAACGGTCCCACTAGTCATCAGTGCATTTACTACCCATGGACGGGCCATTCCACTTTACCACAACATGCTAGAACTCCCCTGATTGCAGCTGGAGTAATTGGTGGGCTCTTCATTCTGGTCATTGTGGGTCTGACATTTGCTGTTTATGTTAGAAGGAAGAGCATCAAAAAGAAAAGAGCCTTGAGAAGATTCTTGGAAACAGAGTTGGTGGAACCATTAACTCCCAGTGGCACAGCACCCAATCAAGCTCAACTTCGTATTTTGAAAGAAACTGAGCTGAAGAGGGTAAAAGTCCTTGGCTCAGGTGCTTTTGGAACGGTTTATAAAGGTATTTGGGTACCTGAAGGAGAAACTGTGAAGATTCCTG | Gene Universal |  |
| pcDNA3.1 ErbB4 JMa/b chimeric CYT-2 –HA, gBlock:  TGGACCGGGTCCTGACAACTGTACAAAGTGCTCTCATTTTAAAGATGGCCCAAACTGTGTGGAAAAATGTCCAGATGGCTTACAGGGGGCAAACAGTTTCATTTTCAAGTATGCTGATCCAGATCGGGAGTGCCACCCATGCCATCCAAACTGCACCCAAGGGTGTAACGGTCCCACTAGTCATGACTGCATTGGCCTGATGGATAGAACTCCCCTGATTGCAGCTGGAGTAATTGGTGGGCTCTTCATTCTGGTCATTGTGGGTCTGACATTTGCTGTTTATGTTAGAAGGAAGAGCATCAAAAAGAAAAGAGCCTTGAGAAGATTCTTGGAAACAGAGTTGGTGGAACCATTAACTCCCAGTGGCACAGCACCCAATCAAGCTCAACTTCGTATTTTGAAAGAAACTGAGCTGAAGAGGGTAAAAGTCCTTGGCTCAGGTGCTTTTGGAACGGTTTATAAAGGTATTTGGGTACCTGAAGGAGAAACTGTGAAG | IDT |  |
| pcDNA3.1 ErbB4 JMb/a chimeric CYT-2 –HA, gBlock: TGGACCGGGTCCTGACAACTGTACAAAGTGCTCTCATTTTAAAGATGGCCCAAACTGTGTGGAAAAATGTCCAGATGGCTTACAGGGGGCAAACAGTTTCATTTTCAAGTATGCTGATCCAGATCGGGAGTGCCACCCATGCCATCCAAACTGCACCCAAGGGTGCATAGGCTCAAGTATTGAAGACTGCATCTACTACCCATGGACGGGCCATTCCACTTTACCACAACATGCTAGAACTCCCCTGATTGCAGCTGGAGTAATTGGTGGGCTCTTCATTCTGGTCATTGTGGGTCTGACATTTGCTGTTTATGTTAGAAGGAAGAGCATCAAAAAGAAAAGAGCCTTGAGAAGATTCTTGGAAACAGAGTTGGTGGAACCATTAACTCCCAGTGGCACAGCACCCAATCAAGCTCAACTTCGTATTTTGAAAGAAACTGAGCTGAAGAGGGTAAAAGTCCTTGGCTCAGGTGCTTTTGGAACGGTTTATAAAGGTATTTGGGTACCTGAAGGAGAAACTGTGAAG | IDT |  |
| pcDNA3.1 ErbB4 JM-a ΔNST CYT-2 -HA, gBlock: TACAATCCAACCACCTTTCAACTGGAGCACAATTTCAATGCAAAGTACACATATGGAGCATTCTGTGTCAAGAAATGTCCACATAACTTTGTGGTAGATTCCAGTTCTTGTGTGCGTGCCTGCCCTAGTTCCAAGATGGAAGTAGAAGAAAATGGGATTAAAATGTGTAAACCTTGCACTGACATTTGCCCAAAAGCTTGTGATGGCATTGGCACAGGATCATTGATGTCAGCTCAGACTGTGGATTCCAGTAACATTGACAAATTCATAAACTGTACCAAGATCAATGGGAATTTGATCTTTCTAGTCACTGGTATTCATGGGGACCCTTACAATGCAATTGAAGCCATAGACCCAGAGAAACTGAACGTCTTTCGGACAGTCAGAGAGATAACAGGTTTCCTGAACATACAGTCATGGCCACCAAACATGACTGACTTCAGTGTTTTTTCTAACCTGGTGACCATTGGTGGAAGAGTACTCTATAGTGGCCTGTCCTTGCTTATCCTCAAGCAACAGGGCATCACCTCTCTACAGTTCCAGTCCCTGAAGGAAATCAGCGCAGGAAACATCTATATTACTGACAACAGCAACCTGTGTTATTATCATACCATTAACTGGACAACACTCTTCAGCACAATCAACCAGAGAATAGTAATCCGGGACAACAGAAAAGCTGAAAATTGTACTGCTGAAGGAATGGTGTGCAACCATCTGTGTTCCAGTGATGGCTGTTGGGGACCTGGGCCAGACCAATGTCTGTCGTGTCGCCGCTTCAGTAGAGGAAGGATCTGCATAGAGTCTTGTAACCTCTATGATGGTGAATTTCGGGAGTTTGAGAATGGCTCCATCTGTGTGGAGTGTGACCCCCAGTGTGAGAAGATGGAAGATGGCCTCCTCACATGCCATGGACCGGGTCCTGACAACTGTACAAAGTGCTCTCATTTTAAAGATGGCCCAAACTGTGTGGAAAAATGTCCAGATGGCTTACAGGGGGCAAACAGTTTCATTTTCAAGTATGCTGATCCAGATCGGGAGTGCCACCCATGCCATCCAAACTGCACCCAAGGGTGTCTCGGTCCCGTTGCTCATGACTGCATTTACTACCCATGGGTGGGCCATGCCGTTTTACCACAACATGCTAGAACTCCCCTGATTGCAGCTGGAGTAATTGGTGGGCTCTTCATTCTGGTCATTGTGGGTCTGACATTTGCTGTTTATGTTAGAAGGAAGAGCATCAAAAAGAAAAGAGCCTTGAGAAGATTCTTGGAAACAGAGTTGGTGGAACCATTAACTCCCAGTGGCACAGCACCCAATCAAGCTCAACTTCGTATTTTGAAAGAAACTGAGCTGAAGAGGGTAAAAGTCCTTGGCTCAGGTGCTTTTGGAACGGTTTATAAAGGTATTTGGGTACCTGAAGGAGAAACTGTGAAGATTCCTGTGGCTATTAAGATTCTTAATGAGACAACTGGTCCCAAGGCAAATGTGGAGTTCATGGAT | IDT |  |
| pcDNA3.1 ErbB4 JMa S630I/H631E CYT-2 –HA, FWRD:  CAAGGGTGTAACGGTCCCACTATTGAAGACTGCATTTAC | Eurofins Genomics |  |
| pcDNA3.1 ErbB4 JMa S630I/H631E CYT-2 –HA, REV:  CCATGGGTAGTAAATGCAGTCTTCAATAGTGGGACCGTTAC | Eurofins Genomics |  |
| pcDNA3.1 ErbB4 JM-a Y984F CYT-2 –HA, FWRD:  GGCTCGAGACCCTCAAAGATTCCTAGTTATTCAGGGT GATGATCG | Oligomer |  |
| pcDNA3.1 ErbB4 JM-a Y984F CYT-2 –HA, REV:  CGATCATCACCCTGAATAACTAGGAATCTTTGAGGGTCTCGAGCC | Oligomer |  |
| pcDNA3.1 ErbB4 JM-a CYT-2 –HA, FWRD: CTGAGGAGTTCTT GGTCCCT CAGGCTTTC | Oligomer |  |
| pcDNA3.1 ErbB4 JM-a CYT-2 –HA, REV: CATCCATC ATATCTTCCAAATCC TCTTCATC | Oligomer |  |
| pcDNA3.1 ErbB4 JM-b ΔS CYT-2 –HA, FWRD: CATCCAAACTGCACCCAAGGGTGCATAGGCGCAGCTATTGAAGAC | Eurofins Genomics |  |
| pcDNA3.1 ErbB4 JM-b ΔS CYT-2 –HA, REV: GTTCTATCCATCAGGCCGATGCAGTCTTCAATAGCTGCGCCTATGCACCCTTG | Eurofins Genomics |  |
| pcDNA3.1 ErbB4 JM-b G627A CYT-2 –HA, GENEFragment: GCCATGGACCGGGTCCTGACAACTGTACAAAGTGCTCTCATTTTAAAGATGGCCCAAACTGTGTGGAAAAATGTCCAGATGGCTTACAGGGGGCAAACAGTTTCATTTTCAAGTATGCTGATCCAGATCGGGAGTGCCACCCATGCCATCCAAACTGCACCCAAGGGTGCATAGCCTCAAGTATTGAAGACTGCATCGGCCTGATGGATAGAACTCCCCTGATTGCAGCTGGAGTAATTGGTGGGCTCTTCATTCTGGTCATTGTGGGTCTGACATTTGCTGTTTATGTTAGAAGGAAGAGCATCAAAAAGAAAAGAGCCTTGAGAAGATTCTTGGAAACAGAGTTGGTGGAACCATTAACTCCCAGTGGCACAGCACCCAATCAAGCTCAACTTCGTATTTTGAAAGAAACTGAGCTGAAGAGGGTAAAAGTCCTTGGCTCAGGTGCTTTTGGAACGGTTTATAAAGGTATTTGGGTACCTGAAGGAGAAACTGTGAAG | Eurofins Genomics |  |
| pcDNA3.1 ErbB4 JM-b D632L CYT-2 –HA, GENEFragment: GCCATGGACCGGGTCCTGACAACTGTACAAAGTGCTCTCATTTTAAAGATGGCCCAAACTGTGTGGAAAAATGTCCAGATGGCTTACAGGGGGCAAACAGTTTCATTTTCAAGTATGCTGATCCAGATCGGGAGTGCCACCCATGCCATCCAAACTGCACCCAAGGGTGCATAGGCTCAAGTATTGAACTCTGCATCGGCCTGATGGATAGAACTCCCCTGATTGCAGCTGGAGTAATTGGTGGGCTCTTCATTCTGGTCATTGTGGGTCTGACATTTGCTGTTTATGTTAGAAGGAAGAGCATCAAAAAGAAAAGAGCCTTGAGAAGATTCTTGGAAACAGAGTTGGTGGAACCATTAACTCCCAGTGGCACAGCACCCAATCAAGCTCAACTTCGTATTTTGAAAGAAACTGAGCTGAAGAGGGTAAAAGTCCTTGGCTCAGGTGCTTTTGGAACGGTTTATAAAGGTATTTGGGTACCTGAAGGAGAAACTGTGAAG | Gene Universal |  |
| pcDNA3.1 ErbB4 JM-b E631D/D632L CYT-2 –HA, GENEFragment: GCCATGGACCGGGTCCTGACAACTGTACAAAGTGCTCTCATTTTAAAGATGGCCCAAACTGTGTGGAAAAATGTCCAGATGGCTTACAGGGGGCAAACAGTTTCATTTTCAAGTATGCTGATCCAGATCGGGAGTGCCACCCATGCCATCCAAACTGCACCCAAGGGTGCATAGGCTCAAGTATTGATCTCTGCATCGGCCTGATGGATAGAACTCCCCTGATTGCAGCTGGAGTAATTGGTGGGCTCTTCATTCTGGTCATTGTGGGTCTGACATTTGCTGTTTATGTTAGAAGGAAGAGCATCAAAAAGAAAAGAGCCTTGAGAAGATTCTTGGAAACAGAGTTGGTGGAACCATTAACTCCCAGTGGCACAGCACCCAATCAAGCTCAACTTCGTATTTTGAAAGAAACTGAGCTGAAGAGGGTAAAAGTCCTTGGCTCAGGTGCTTTTGGAACGGTTTATAAAGGTATTTGGGTACCTGAAGGAGAAACTGTGAAG | Gene Universal |  |
| pcDNA3.1 ErbB4 JM-b E631L/D632L CYT-2 –HA, GENEFragment: GCCATGGACCGGGTCCTGACAACTGTACAAAGTGCTCTCATTTTAAAGATGGCCCAAACTGTGTGGAAAAATGTCCAGATGGCTTACAGGGGGCAAACAGTTTCATTTTCAAGTATGCTGATCCAGATCGGGAGTGCCACCCATGCCATCCAAACTGCACCCAAGGGTGCATAGGCTCAAGTATTCTACTCTGCATCGGCCTGATGGATAGAACTCCCCTGATTGCAGCTGGAGTAATTGGTGGGCTCTTCATTCTGGTCATTGTGGGTCTGACATTTGCTGTTTATGTTAGAAGGAAGAGCATCAAAAAGAAAAGAGCCTTGAGAAGATTCTTGGAAACAGAGTTGGTGGAACCATTAACTCCCAGTGGCACAGCACCCAATCAAGCTCAACTTCGTATTTTGAAAGAAACTGAGCTGAAGAGGGTAAAAGTCCTTGGCTCAGGTGCTTTTGGAACGGTTTATAAAGGTATTTGGGTACCTGAAGGAGAAACTGTGAAG | Gene Universal |  |
| pcDNA3.1 ErbB4 JM-b E631N/D632L CYT-2 –HA, FWRD:  GCATAGGCTCAAGTATTAATCTCTGCATCGG | Eurofins Genomics |  |
| pcDNA3.1 ErbB4 JM-b E631N/D632L CYT-2 –HA, REV:  CCATCAGGCCGATGCAGAGGTTAATACTTG | Eurofins Genomics |  |
| pcDNA3.1 ErbB4 JM-b E631Q/D632L CYT-2 –HA, GENEFragment:  GCCATGGACCGGGTCCTGACAACTGTACAAAGTGCTCTCATTTTAAAGATGGCCCAAACTGTGTGGAAAAATGTCCAGATGGCTTACAGGGGGCAAACAGTTTCATTTTCAAGTATGCTGATCCAGATCGGGAGTGCCACCCATGCCATCCAAACTGCACCCAAGGGTGCATAGGCTCAAGTATTCAACTCTGCATCGGCCTGATGGATAGAACTCCCCTGATTGCAGCTGGAGTAATTGGTGGGCTCTTCATTCTGGTCATTGTGGGTCTGACATTTGCTGTTTATGTTAGAAGGAAGAGCATCAAAAAGAAAAGAGCCTTGAGAAGATTCTTGGAAACAGAGTTGGTGGAACCATTAACTCCCAGTGGCACAGCACCCAATCAAGCTCAACTTCGTATTTTGAAAGAAACTGAGCTGAAGAGGGTAAAAGTCCTTGGCTCAGGTGCTTTTGGAACGGTTTATAAAGGTATTTGGGTACCTGAAGGAGAAACTGTGAAG | Eurofins Genomics |  |
| pcDNA3.1 ErbB4 JM-b Y974F CYT-2 –HA, FWRD: CAAAGATTCCTAGTTATTCAGGGTGATGATCGTATGAAGCTTCCCA | Eurofins Genomics |  |
| pcDNA3.1 ErbB4 JM-b Y974F CYT-2 –HA, REV: GAATAACTAGGAATCTTTGAGGGTCTCGAGCCATCCTTGAAAACTC | Eurofins Genomics |  |
| pcDNA3.1 ErbB4 JM-b Y1012F CYT-2 –HA, FWRD:  GAGGAGTTCTTGGTCCCTCAGGCTTTCAACATCCC | Eurofins Genomics |  |
| pcDNA3.1 ErbB4 JM-b Y1012F CYT-2 –HA, REV:  GAGGGACCAAGAACTCCTCAGCATCCATCATATCTTCC | Eurofins Genomics |  |
| pcDNA3.1 ErbB4 JM-b K741R CYT-2 –HA, FWRD:  GGCTATTGCTATTCTTAATGAGACAACTGGTCCCAAG | Eurofins Genomics |  |
| pcDNA3.1 ErbB4 JM-b K741R CYT-2 –HA, REV:  GTCTCATTAAGAATAGCAATAGCCACAGGAATCTTCACAG | Eurofins Genomics |  |
| pDest-N1-TYK2 K930I –eGFP, FWRD: GCGGTGATAGCCCTCAAGGCAGACTG | Eurofins Genomics |  |
| pDest-N1-TYK2 K930I –eGFP, REV: GAGGGCTATCACCGCCACCATCTCG | Eurofins Genomics |  |
| px330 Neo, FWRD:  GCGGAGATATCCAGCTGTGGAATGTGTG | Eurofins Genomics |  |
| px330 Neo, REV:  GATATGATATCCCTGAGGCTATGGCAGGG | Eurofins Genomics |  |
| px330 Neo TYK2 KO2 CRISPR, FWRD: CACCGCCTTCGGAACGTCTTCCGC | Eurofins Genomics |  |
| px330 Neo TYK2 KO2 CRISPR, REV: AAACGCGGAAGACGTTCCGAAGGC | Eurofins Genomics |  |
| px330 Neo TYK2 KO4 CRISPR, FWRD: CACCGAACCGGCTGTGTACCGTTG | Eurofins Genomics |  |
| px330 Neo TYK2 KO4 CRISPR, REV: AAACCAACGGTACACAGCCGGTTC | Eurofins Genomics |  |
| qPCR primer *β-actin* FWRD:  ATCTGGCACCACACCTTCTACAAT | Oligomer |  |
| qPCR primer *β-actin* REV:  CCGTCACCGGAGTCCATCA | Oligomer |  |
| qPCR probe *β-actin*-FAM:  TGACCCAGATCATGTTTGAGACCTTCAACAC | Eurogentec |  |
| qPCR primer *ErbB4* JM-a /JM-b, JM-1 FWRD:  ttgccatccaaactgcacc | Eurogentec |  |
| qPCR primer *ErbB4* JM-a /JM-b, JM-2 REV:  tccaatgactccggctgc-3 | Eurogentec |  |
| qPCR Probe: *ErbB4* JM-a-FAM: CAT GGA CGG GCC ATT CCA CTT TAC CA | Eurogentec |  |
| qPCR Probe: *ErbB4* JM-b-FAM:  ttc aag cat tga aga ctg cat cgg cct | Eurogentec |  |
| pBABE-puro ErbB4 JM-a V675A CYT-2 –HA | Described here |  |
| pDest-eGFP-N1 | Addgene | 31796 |
| pDest-JAK1-eGFP-N1 | Described here |  |
| pDest-JAK2-eGFP-N1 | Described here |  |
| pDest-JAK3-eGFP-N1 | Described here |  |
| pDest-TYK2-eGFP-N1 | Described here |  |
| pDest-TYK2 K930I-eGFP-N1 | Described here |  |
| pcDNA3.1 Hyg(+) | ^1^ |  |
| pcDNA3.1 ErbB1-HA | ^2^ |  |
| pcDNA3.1 ErbB2-HA | ^2^ |  |
| pcDNA3.1 ErbB3-HA | ^2^ |  |
| pcDNA3.1 ErbB4 JM-a CYT-2 –eGFP Hyg(+) | ^1^ |  |
| pcDNA3.1 ErbB4 JM-a CYT-2 –HA Hyg(+) | ^1^ |  |
| pcDNA3.1 ErbB4 JM-a CYT-2 –MYC Hyg(+) | ^3^ |  |
| pcDNA3.1 ErbB4 JM-a ΔNST CYT-2 –HA Hyg(+) | Described here |  |
| pcDNA3.1 ErbB4 JM-a G627A CYT-2 –HA Hyg(+) | Described here |  |
| pcDNA3.1 ErbB4 JM-a S630I/H631E CYT-2 –HA Hyg(+) | Described here |  |
| pcDNA3.1 ErbB4 JM-a H631E CYT-2 –HA Hyg(+) | Described here |  |
| pcDNA3.1 ErbB4 JM-a H631K CYT-2 –HA Hyg(+) | Described here |  |
| pcDNA3.1 ErbB4 JM-a H631N CYT-2 –HA Hyg(+) | Described here |  |
| pcDNA3.1 ErbB4 JM-a H631Q CYT-2 –HA Hyg(+) | Described here |  |
| pcDNA3.1 ErbB4 JM-a H631R CYT-2 –HA Hyg(+) | Described here |  |
| pcDNA3.1 ErbB4 JM-a D632E CYT-2 –HA Hyg(+) | Described here |  |
| pcDNA3.1 ErbB4 JM-a D632H CYT-2 –HA Hyg(+) | Described here |  |
| pcDNA3.1 ErbB4 JM-a D632L CYT-2 –HA Hyg(+) | Described here |  |
| pcDNA3.1 ErbB4 JM-a D632N CYT-2 –HA Hyg(+) | Described here |  |
| pcDNA3.1 ErbB4 JM-a D632Q CYT-2 –HA Hyg(+) | Described here |  |
| pcDNA3.1 ErbB4 JM-a Y984F CYT-2 –HA Hyg(+) | Described here |  |
| pcDNA3.1 ErbB4 JM-a Y1022F CYT-2 –HA Hyg(+) | Described here |  |
| pcDNA3.1 ErbB4 JMa/b chimeric CYT-2 –HA Hyg(+) | Described here |  |
| pcDNA3.1 ErbB4 JMb/a chimeric CYT-2 –HA Hyg(+) | Described here |  |
| pcDNA3.1 ErbB4 JM-b CYT-2 –HA Hyg(+) | ^1^ |  |
| pcDNA3.1 ErbB4 JM-b CYT-2 –MYC Hyg(+) | ^1^ |  |
| pcDNA3.1 ErbB4 JM-b ΔS CYT-2 –HA Hyg(+) | Described here |  |
| pcDNA3.1 ErbB4 JM-b G627A CYT-2 –HA Hyg(+) | Described here |  |
| pcDNA3.1 ErbB4 JM-b D632L CYT-2 –HA Hyg(+) | Described here |  |
| pcDNA3.1 ErbB4 JM-b E631D/D632L CYT-2 –HA Hyg(+) | Described here |  |
| pcDNA3.1 ErbB4 JM-b E631L/D632L CYT-2 –HA Hyg(+) | Described here |  |
| pcDNA3.1 ErbB4 JM-b E631N/D632L CYT-2 –HA Hyg(+) | Described here |  |
| pcDNA3.1 ErbB4 JM-b E631Q/D632L CYT-2 –HA Hyg(+) | Described here |  |
| pcDNA3.1 ErbB4 JM-b K741R CYT-2 –HA Hyg(+) | Described here |  |
| pcDNA3.1 ErbB4 JM-b Y974F CYT-2 –HA Hyg(+) | Described here |  |
| pcDNA3.1 ErbB4 JM-b Y1012F CYT-2 –HA Hyg(+) | Described here |  |
| pDONR223-JAK1 | Addgene | 23932 |
| pDONR223-JAK2 | Addgene | 23915 |
| pDONR223-JAK3 | Addgene | 23944 |
| pDONR223-TYK2 | Addgene | 23908 |
| pLX302 EphA2-V5 | ^2^ |  |
| pLX302 EphA7-V5 | ^2^ |  |
| pLX302 FGFR3-V5 | ^2^ |  |
| pLX302 INSRR-V5 | ^2^ |  |
| pLX302 TIE1-V5 | ^2^ |  |
| pLX302 TYRO3-V5 | ^2^ |  |
| pMD2.G (env) | Addgene | 12259 |
| pMDLg/pRRE (gag, pol, RRE) | Addgene | 12251 |
| pME18S-STAT5a | ^4^ |  |
| pME18S-STAT5b | ^4^ |  |
| pRc/CMV STAT5b Y699F | ^5^ |  |
| pRc/CMV STAT5b Y699F/Y724F | ^5^ |  |
| pRc/CMV STAT5b Y699F/Y739F | ^5^ |  |
| pRSV-Rev (Rev) | Addgene | 12253 |
| pX330-U6-Chimeric_BB-CBh-hSpCas9 | Addgene | 42230 |
| px330 Neo | Described here |  |
| px330 Neo TYK2 KO2 CRISPR | Described here |  |
| px330 Neo TYK2 KO4 CRISPR | Described here |  |
| **siRNAs and shRNAs** |  |  |
| siRNA control: AllStars Negative Control siRNA | Qiagen | SI03650318 |
| siRNA mm_STAT5a | Ambion | s74458 |
| siRNA mm_STAT5a | Ambion | 69369 |
| siRNA mm_STAT5b_3 | Qiagen | SI04943183 |
| siRNA mm_STAT5b_4 | Qiagen | SI00201194 |
| siRNA TYK2: Hs_TYK2_5 FlexiTube siRNA | Qiagen | SI02223221 |
| siRNA TYK2: Hs_TYK2_3 FlexiTube siRNA | Qiagen | SI00050918 |
| FlexiTube GeneSolution GS3717 for JAK2 | Qiagen | 1027416 |
| **Synthetic Peptides** |  |  |
| JMa: SGSGCTQGSNGPTSHDC, N-terminal biotin, C-terminal amide, cyclized | JPT | custom |
| JMb: SGSGCTQGSIGSSIEDC, N-terminal biotin, C-terminal amide, cyclized | JPT | custom |
| **Arrays** |  |  |
| Membrane Lipid Arrays | Echelon Bioscience | P-6003 |
| Consortium for Functional Glycomics (CFG) mammalian glycan array | CFG's Protein-Glycan Interaction Core |  |
| **Experimental Models: Cell Lines** |  |  |
| COS-7 | ^1^ |  |
| Phoenix Ampho HEK293 | ATCC |  |
| HC11 | ^6^ |  |
| HC11 pBABE-puro | ^6^ |  |
| HC11 pBABE-puro ErbB4 JM-a CYT-1 | Described here |  |
| HC11 pBABE-puro ErbB4 JM-a CYT-2 | ^6^ |  |
| HC11 pBABE-puro ErbB4 JM-a CYT-2 -HA | Described here |  |
| HC11 pBABE-puro ErbB4 JM-a V675A CYT-2 -HA | Described here |  |
| HC11 pBABE-puro ErbB4 JM-b CYT-1 | Described here |  |
| HC11 pBABE-puro ErbB4 JM-b CYT-2 | ^6^ |  |
| MCF7 | ECACC | ECACC 86012803 |
| MDA-MB-468 | ^7^ |  |
| MDA-MB-468 pBABE-puro | Described here |  |
| MDA-MB-468 pBABE-puro ErbB4 JM-a CYT-1 | ^8^ |  |
| MDA-MB-468 pBABE-puro ErbB4 JM-a CYT-2 | Described here |  |
| MDA-MB-468 pBABE-puro ErbB4 JM-b CYT-1 | Described here |  |
| MDA-MB-468 pBABE-puro ErbB4 JM-b CYT-2 | Described here |  |
| **Three-dimensional structures** |  |  |
| X-ray structure of extracellular region of human ErbB4 dimer with bound NRG-1β, PDB code 3U7U | ^9^ |  |
| NMR structure of human ErbB4 transmembrane helix dimer, PDB code 2LCX | ^10^ |  |
| **Datasets and data resources** |  |  |
| Compartments | ^11^ |  |
| Gene Ontology | ^12,13^ |  |
| Molecular Signatures Database (MSigDB v7.5.1) | ^14^ |  |
| Protein Data Bank (PDB) | ^15^ |  |
| PSICQUIC | ^16^ |  |
| STRING | ^17^ |  |
| **Software and algorithms** |  |  |
| AMBER package version 2018 | ^18^ |  |
| Berendsen coupling scheme | ^19^ |  |
| Bodil | ^20^ |  |
| Chimera | ^21^ |  |
| Colocalization algorithm 2011 Villalta et al. | ^22^ |  |
| CPPTRAJ | ^23^ |  |
| ff14SB force field | ^24^ |  |
| FiJi | ^25^ |  |
| FlashLFQ | ^26^ |  |
| GraphPad Prism | GraphPad Software |  |
| Image Studio Lite | LI-COR Biosciences |  |
| IncuCyte ZOOM Software | Essen Bioscience |  |
| Matlab R2016a | Mathworks |  |
| Mascot | Matrix Science |  |
| MetaMorpheus | ^27^ |  |
| The Particle Mesh Ewald algorithm | ^28^ |  |
| PANTHER overrepresentation test | ^29^ |  |
| R | ^30^ |  |
| TIP3P water box | ^31^ |  |
| VMD | ^32^ |  |

*W: western analysis, IF: immunofluorescence, IP: immunoprecipitation, PLA: proximity ligation assay

**REFERENCES**

1. Maatta, J. A. *et al.* Proteolytic Cleavage and Phosphorylation of a Tumor-associated ErbB4 Isoform Promote Ligand-independent Survival and Cancer Cell Growth. *Mol Biol Cell* **17**, 67–79 (2005).

2. Merilahti, J. A. M., Ojala, V. K., Knittle, A. M., Pulliainen, A. T. & Elenius, K. Genome-wide screen of gamma-secretase–mediated intramembrane cleavage of receptor tyrosine kinases. *Mol Biol Cell* **28**, 3123–3131 (2017).

3. Sundvall, M. *et al.* Differential nuclear localization and kinase activity of alternative ErbB4 intracellular domains. *Oncogene* **26**, 6905–14 (2007).

4. Pircher, T. J. *et al.* Mitogen-activated protein kinase kinase inhibition decreases growth hormone stimulated transcription mediated by STAT5. *Mol Cell Endocrinol* **133**, 169–176 (1997).

5. Kabotyanski, E. B. & Rosen, J. M. Signal transduction pathways regulated by prolactin and Src result in different conformations of activated Stat5b. *J Biol Chem* **278**, 17218–27 (2003).

6. Sundvall, M. *et al.* Protein Inhibitor of Activated STAT3 (PIAS3) Protein Promotes SUMOylation and Nuclear Sequestration of the Intracellular Domain of ErbB4 Protein. *Journal of Biological Chemistry* **287**, 23216–23226 (2012).

7. Tvorogov, D. *et al.* Somatic mutations of ErbB4: selective loss-of-function phenotype affecting signal transduction pathways in cancer. *J Biol Chem* **284**, 5582–91 (2009).

8. Paatero, I. *et al.* Hypoxia-inducible factor-1α induces ErbB4 signaling in the differentiating mammary gland. *J Biol Chem* **289**, 22459–69 (2014).

9. Liu, P. *et al.* A single ligand is sufficient to activate EGFR dimers. *Proceedings of the National Academy of Sciences* **109**, 10861–10866 (2012).

10. Bocharov, E. V., Mineev, K. S., Goncharuk, M. V. & Arseniev, A. S. Structural and thermodynamic insight into the process of “weak” dimerization of the ErbB4 transmembrane domain by solution NMR. *Biochimica et Biophysica Acta (BBA) - Biomembranes* **1818**, 2158–2170 (2012).

11. Binder, J. X. *et al.* COMPARTMENTS: unification and visualization of protein subcellular localization evidence. *Database* **2014**, bau012–bau012 (2014).

12. Ashburner, M. *et al.* Gene Ontology: tool for the unification of biology. *Nat Genet* **25**, 25–29 (2000).

13. Carbon, S. *et al.* The Gene Ontology resource: Enriching a GOld mine. *Nucleic Acids Res* **49**, D325–D334 (2021).

14. Subramanian, A. *et al.* Gene set enrichment analysis: A knowledge-based approach for interpreting genome-wide expression profiles. *Proc Natl Acad Sci U S A* **102**, 15545–15550 (2005).

15. Berman, H. M. *et al.* The protein data bank. *Nucleic Acids Res* **28**, 235–242 (2000).

16. del-Toro, N. *et al.* A new reference implementation of the PSICQUIC web service. *Nucleic Acids Res* **41**, W601–W606 (2013).

17. Szklarczyk, D. *et al.* STRING v11: Protein-protein association networks with increased coverage, supporting functional discovery in genome-wide experimental datasets. *Nucleic Acids Res* **47**, D607–D613 (2019).

18. D.A. Case, I.Y. Ben-Shalom, S.R. Brozell, D.S. Cerutti, T.E. Cheatham, III, V.W.D. Cruzeiro, T. A. D. *et al.* AMBER 2018. Preprint at (2018).

19. Berendsen, H. J. C., Postma, J. P. M., van Gunsteren, W. F., DiNola, A. & Haak, J. R. Molecular dynamics with coupling to an external bath. *J Chem Phys* **81**, 3684–3690 (1984).

20. Lehtonen, J. V. *et al.* BODIL: a molecular modeling environment for structure-function analysis and drug design. *J Comput Aided Mol Des* **18**, 401–419 (2004).

21. Pettersen, E. F. *et al.* UCSF Chimera - A visualization system for exploratory research and analysis. *J Comput Chem* **25**, 1605–1612 (2004).

22. Villalta, J. I. *et al.* New Algorithm to Determine True Colocalization in Combination with Image Restoration and Time-Lapse Confocal Microscopy to Map Kinases in Mitochondria. *PLoS One* **6**, e19031 (2011).

23. Roe, D. R. & Cheatham, T. E. PTRAJ and CPPTRAJ: Software for Processing and Analysis of Molecular Dynamics Trajectory Data. *J Chem Theory Comput* **9**, 3084–3095 (2013).

24. Maier, J. A. *et al.* ff14SB: Improving the Accuracy of Protein Side Chain and Backbone Parameters from ff99SB. *J Chem Theory Comput* **11**, 3696–3713 (2015).

25. Schindelin, J. *et al.* Fiji: An open-source platform for biological-image analysis. *Nature Methods* vol. 9 676–682 Preprint at https://doi.org/10.1038/nmeth.2019 (2012).

26. Millikin, R. J., Solntsev, S. K., Shortreed, M. R. & Smith, L. M. Ultrafast Peptide Label-Free Quantification with FlashLFQ. *J Proteome Res* **17**, 386–391 (2018).

27. Solntsev, S. K., Shortreed, M. R., Frey, B. L. & Smith, L. M. Enhanced Global Post-translational Modification Discovery with MetaMorpheus. *J Proteome Res* **17**, 1844–1851 (2018).

28. Essmann, U. *et al.* A smooth particle mesh Ewald method. *J Chem Phys* **103**, 8577–8593 (1995).

29. Mi, H., Muruganujan, A., Ebert, D., Huang, X. & Thomas, P. D. PANTHER version 14: more genomes, a new PANTHER GO-slim and improvements in enrichment analysis tools. *Nucleic Acids Res* **47**, D419 (2019).

30. R Core Team. R Core Team (2017). R: A language and environment for statistical computing. *R Foundation for Statistical Computing, Vienna, Austria. URL http://www.R-project.org/.* R Foundation for Statistical Computing (2017).

31. Jorgensen, W. L., Chandrasekhar, J., Madura, J. D., Impey, R. W. & Klein, M. L. Comparison of simple potential functions for simulating liquid water. *J Chem Phys* **79**, 926–935 (1983).

32. Humphrey, W., Dalke, A. & Schulten, K. VMD: Visual molecular dynamics. *J Mol Graph* **14**, 33–38 (1996).
