# Supplementary material for: An extracellular receptor tyrosine kinase motif orchestrating intracellular STAT activation
Source: Nat Commun. 2022 Nov 14;13:6953. doi: 10.1038/s41467-022-34539-4 (PMC9663514; doi:10.1038/s41467-022-34539-4)
Supplement: Supplementary file 14 — Reporting Summary [file 41467_2022_34539_MOESM14_ESM.pdf]

nature research

Corresponding author(s): Klaus Elenius

Last updated by author(s): Nov 8, 2022

Reporting Summary

Nature Research wishes to improve the reproducibility of the work that we publish. This form provides structure for consistency and transparency in reporting. For further information on Nature Research policies, see Authors & Referees and the Editorial Policy Checklist .

Statistics

For all statistical analyses, confirm that the following items are present in the figure legend, table legend, main text, or Methods section.

n/a	Confirmed	
☐	☒	The exact sample size (*n*) for each experimental group/condition, given as a discrete number and unit of measurement
☐	☒	A statement on whether measurements were taken from distinct samples or whether the same sample was measured repeatedly
☐	☒	The statistical test(s) used AND whether they are one- or two-sided<br>*Only common tests should be described solely by name; describe more complex techniques in the Methods section.*
☐	☒	A description of all covariates tested
☐	☒	A description of any assumptions or corrections, such as tests of normality and adjustment for multiple comparisons
☐	☒	A full description of the statistical parameters including central tendency (e.g. means) or other basic estimates (e.g. regression coefficient) AND variation (e.g. standard deviation) or associated estimates of uncertainty (e.g. confidence intervals)
☐	☒	For null hypothesis testing, the test statistic (e.g. *F*, *t*, *r*) with confidence intervals, effect sizes, degrees of freedom and *P* value noted<br>*Give P values as exact values whenever suitable.*
☒	☐	For Bayesian analysis, information on the choice of priors and Markov chain Monte Carlo settings
☒	☐	For hierarchical and complex designs, identification of the appropriate level for tests and full reporting of outcomes
☐	☒	Estimates of effect sizes (e.g. Cohen's *d*, Pearson's *r*), indicating how they were calculated

Our web collection on statistics for biologists contains articles on many of the points above.

Software and code

Policy information about availability of computer code

Data collection	-

Data analysis

For image analysis FiJi (Schindelin, J. et al. Fiji: An open-source platform for biological-image analysis. Nature Methods 9, 676–682 (2012), versions 1.50 and 1.51) was used. For quantification of Western blot analyses, Image Studio Lite, version 5.2 (LI-COR Biosciences, NE, USA) was used. For live-imaging analysis, Incucyte ZOOM 2018b (Sartorius, Germany) software was used. For mass spectrometry data analysis FlashLFQ (Millikin, R. J., Solntsev, S. K., Shortreed, M. R. & Smith, L. M. Ultrafast Peptide Label-Free Quantification with FlashLFQ. J. Proteome Res. 17, 386–391 (2018), version 1.1.2) and Metamorpheus v. 0.0.304 (Solntsev, S. K., Shortreed, M. R., Frey, B. L. & Smith, L. M. Enhanced Global Posttranslational Modification Discovery with MetaMorpheus. J. Proteome Res. 17, 1844–1851 (2018)) were used. For standard normalization, differential expression, complex analysis and statistical analysis standard functions in Matlab 2016a (Mathworks, MA, USA) and R versions 3.0.3-3.6.2 (R Core Team. R Core Team (2017). R: A language and environment for statistical computing. R Found. Stat. Comput. Vienna, Austria. URL http://www.R-project.org/. R Foundation for Statistical Computing (2017)) were used and the methods are described in detail in the materials and methods section, making them easily reproducible with any statistical program. For visualiztion and statistical testing GraphPad Prism (GraphPad software, San Diego, CA, USA, v.8.3.0 - v. 9.4.1) was additionally used. The final R code used to find JM sequence motifs in the eJM sequence of receptor tyrosine kinases is available through github: https://github.com/kvaparanta/JM_motif_algorithm. The structural models were built and visualized with Modeller program version 9.9 (Šali, A. & Blundell, T. L. Comparative protein modelling by satisfaction of spatial restraints. J. Mol. Biol. 234, 779–815 (1993)), Chimera versoin 1.13.1 (Pettersen, E. F. et al. UCSF Chimera - A visualization system for exploratory research and analysis. J. Comput. Chem. 25, 1605–1612 (2004).), Maestro version 2019-1 (Schrödinger LLC, New York, N.) and Bodil version 0.8 (Lehtonen, J. V. et al. BODIL: a molecular modeling environment for structure-function analysis and drug design. J. Comput. Aided. Mol. Des. 18, 401–419 (2004).). For molecular dynamics simulations CHARMM-GUI web server version 3.0 (Lee, J. et al. CHARMM-GUI Input Generator for NAMD, GROMACS, AMBER, OpenMM, and CHARMM/OpenMM Simulations Using the CHARMM36 Additive Force Field. J. Chem. Theory Comput. 12, 405–413 (2016).), Amber program version 18 (D.A. Case, I.Y. Ben-Shalom, S.R. Brozell, D.S. Cerutti, T.E. Cheatham, III, V.W.D. Cruzeiro, T. A. D. et al. AMBER 2018. (2018)), CPPTRAJ version 16 (Roe, D. R. & Cheatham, T. E. PTRAJ and CPPTRAJ: Software for Processing and Analysis of Molecular Dynamics Trajectory Data. J. Chem. Theory Comput. 9, 3084–3095 (2013)) and VMD version 1.9.3 (Humphrey, W., Dalke, A. & Schulten, K. VMD: Visual molecular dynamics. J. Mol. Graph. 14, 33–38 (1996).).

For manuscripts utilizing custom algorithms or software that are central to the research but not yet described in published literature, software must be made available to editors/reviewers. We strongly encourage code deposition in a community repository (e.g. GitHub). See the Nature Research guidelines for submitting code & software for further information.

Data

Policy information about availability of data

All manuscripts must include a data availability statement. This statement should provide the following information, where applicable:

- Accession codes, unique identifiers, or web links for publicly available datasets
- A list of figures that have associated raw data
- A description of any restrictions on data availability

The raw MS proteomics data have been deposited to the ProteomeXchange Consortium via the PRIDE56 and Panorama Public57 partner repositories with the data set identifiers PXD017783, PXD026546, PXD026617, and PXD026617. The protein abundances derived from the analyzed MS proteomics data are additionally provided as Supplementary Data 1-4 and 9. The raw data from the glycan screen are provided as a Supplementary Data 8. The raw data on the molecular dynamics simulations have been supplied to Mendely data: http://dx.doi.org/10.17632/y7b9mgdhrb.1. The ErbB4 structures 3U7U (http://dx.doi.org/10.2210/pdb3u7u/pdb) and 2LCX (http://doi.org/10.2210/pdb2LCX/pdb), can be accessed through PDB. The raw data on RTK interactomes can be accessed through MassIVE with dataset ID MSV000087816 (https://massive.ucsd.edu/ProteoSAFe/dataset.jsp?task=b45c797348cc484baff3e8100e4373e8). The subcellular locations on COMPARTMENTS (https://compartments.jensenlab.org/), protein-protein interactions on STRING (https://string-db.org/) and PSIQUIC (http://www.ebi.ac.uk/Tools/webservices/psicquic/view/main.xhtml) and sequence information in Uniprot (https://www.uniprot.org/) can be accessed through the respective website of the database. Source data on Figures 1-6 and Supplementary Figures 1-13 are provided with this paper. Additional source data that support the findings of this study are available from the corresponding author upon reasonable request.The additional source data was not deposited to a public repository since the public repositories do not support the deposition of over 1 TB of data of various data file types.

The subcellular localization data in Compartments database were accessed through their website (Janos X. Binder, Sune Pletscher-Frankild, Kalliopi Tsafou, Christian Stolte, Seán I. O'Donoghue, Reinhard Schneider, Lars Juhl Jensen, COMPARTMENTS: unification and visualization of protein subcellular localization evidence, Database, Volume 2014, 2014, bau012, https://compartments.jensenlab.org ; accessed 19.4.2021). The protein-protein interaction information on PSIQUIC database was accessed through a R vignette (Shannon P (2016). PSICQUIC: Proteomics Standard Initiative Common QUery InterfaCe. R package version 1.28.0.). The protein-protein interaction information on the STRING database were accessed through their website (Szklarczyk et al. Nucleic acids research 47.D1 (2018): D607-D613.2, https://string-db.org/, v.11.0).The sequence information of human receptor tyrosine kinases was accessed through the Uniprot website (The UniProt Consortium UniProt: the universal protein knowledgebase. Nucleic Acids Res. 46: 2699 (2018), https://www.uniprot.org/, release-2018-03). The protein structure information was accessed through the Protein Databank website (H.M. Berman, J. Westbrook, Z. Feng, G. Gilliland, T.N. Bhat, H. Weissig, I.N. Shindyalov, P.E. Bourne. (2000) The Protein Data Bank Nucleic Acids Research, 28: 235-242, rcsb.org, accessed 5.12.2017).

Field-specific reporting

Please select the one below that is the best fit for your research. If you are not sure, read the appropriate sections before making your selection.

☒ Life sciences ☐ Behavioural & social sciences ☐ Ecological, evolutionary & environmental sciences

For a reference copy of the document with all sections, see nature.com/documents/nr-reporting-summary-flat.pdf

Life sciences study design

All studies must disclose on these points even when the disclosure is negative.

Sample size

The study was conducted with cell lines, which is why the traditional sense of sample size does not apply. For quantitative studies, the amount of analyzed samples was determined by how many replicates were needed to gain a significant P-value (under 0.05) for observed differences.

	For data following the normal distribution a minimum of 3 and for non-normal data a minimum of 4 samples were needed for a statistical signficant results. If significant overlap between the sample values of different conditions was observed, additional repetitions were conducted to reach statistical significance.
Data exclusions	Data were excluded from analyses due to technical concerns. In live-imaging experiments, cell plate wells with a significantly different confluence in the beginning of the experiments were excluded. Western blot bands with smeared wells or unfortunate air bubbles or very low signal against the background were excluded. In immunofluorescence imaging experiments, cells that had highly irregular morphology against other cells in the slide were excluded. Experiments with failed positive and/or negative controls were excluded.
Replication	The findings were replicated in different cell lines to ensure reproducibility. All experiments were replicated at least twice in the exact same setting to ensure reproducibility. Different approaches to show the same phenomenon were applied to ensure reproducibility. All attempts at replication that were not successful were due to technical concerns.
Randomization	Randomization of samples to group allocation is not relevant to the study. The experimental model used was cell lines. By using cell lines the background of each treatment is identical.
Blinding	Blinding to group allocation during collection or analysis was not feasible since most experiments were conducted independently by one investigator and the sample order needed to be recorded for accurate interpretation of results (western analyses, immunofluorescence analyses). The experiments conducted with more than one investigator were blinded by not divulging the details of the samples to the second investigator running the analyses (mass spectrometry experiments).

Reporting for specific materials, systems and methods

We require information from authors about some types of materials, experimental systems and methods used in many studies. Here, indicate whether each material, system or method listed is relevant to your study. If you are not sure if a list item applies to your research, read the appropriate section before selecting a response.

Materials & experimental systems

n/a	Involved in the study
☐	☒ Antibodies
☐	☒ Eukaryotic cell lines
☒	☐ Palaeontology
☒	☐ Animals and other organisms
☒	☐ Human research participants
☒	☐ Clinical data

Methods

n/a	Involved in the study
☒	☐ ChIP-seq
☒	☐ Flow cytometry
☒	☐ MRI-based neuroimaging

Antibodies

Antibodies used

Mouse monoclonal anti-β-actin (AC-74) , Sigma , Catalog no: A5441 , Lot no: 026M478OV, 1:2000 dilution in western analyses ;
Goat polyclonal anti-Actin (I-19) , Santa Cruz , Catalog no: sc-1616 , Lot no: (Antibody not available anymore), 1:1000 dilution in western analyses;
Mouse monoclonal anti-β1-integrin (4B7R) , Santa Cruz , Catalog no: sc-9970 , Lot no: J129, 1:100 dilution in immunofluorescence analyses;
Mouse monoclonal anti-β1-integrin (K-20) , Santa Cruz, Catalog no: sc-18887, Lot no: #G1713, 1:100 dilution in immunofluorescence analyses;
Rabbit monoclonal anti-β1-integrin (EP1041Y) , Abcam , Catalog no: ab52971 , Lot no: 927161, 1:1000 dilution in western analyses;
Rat monoclonal anti-β1-integrin (Mab 13) , BD Pharmingen , Catalog no: 552828 , Lot no: 8054, 1ug per 1mg of protein in immunoprecipitation ;
Rabbit monoclonal anti-β4-integrin (D8P6C), Cell Signaling, Catalog no: #14803, Lot no: 1, 1:1000 dilution in western analyses;
Rabbit polyclonal anti-β-Tubulin (H-235) , Santa Cruz , Catalog no: sc-9104 , Lot no: (Antibody not available anymore), 1:1000 dilution in western analyses;
Mouse monoclonal anti-β-Tubulin (SAP.4G5) , Sigma , Catalog no: T7816 , Lot no: 025M4776V, 1:2000 dilution in western analyses;
Rabbit polyclonal EGF Receptor Antibody, Cell Signaling, Catalog no: #2232, Lot no: 16, 1:1000 dilution in western analyses;
Rabbit monoclonal Phospho-EGF Receptor (Tyr1068) (D7A5), Cell Signaling, Catalog no: #3777, Lot no: 10, 1:500 dilution in western analyses;
Rabbit polyclonal ErbB2 Antibody (C-18) Santa Cruz, Catalog no: sc-284, Lot no: #H2411, 1:1000 dilution in western analyses;
Rabbit monoclonal Phospho-HER2/ErbB2 (Tyr1248) Antibody, Cell Signaling, Catalog no: #2247, Lot no: 9, 1:500 dilution in western analyses;
Rabbit monoclonal anti-ErbB4 (E200) , Abcam , Catalog no: ab32375 , Lot no: GR3869848-2, 1:1000 and 1:100 dilution in western and immunofluorescence analyses respectively;
Mouse monoclonal anti-ErbB4 (HFR-1) , Abcam , Catalog no: ab19391 , Lot no: GR1559993330-8, 1:50 dilution in immunofluorscence analyses ;
Rabbit monoclonal anti- Phospho-HER4/ErbB4 (Tyr1284) (21A9) , Cell Signaling , Catalog no: #4757 , Lot no: 6, 1:500 dilution in western analyses;
Rabbit polyclonal anti-GFP , Abcam , Catalog no: ab6556 , Lot no: GR3216972-1, 2ug per 1 mg of protein for

immunoprecipitation, 1:1000 dilution in western analyses ;
Mouse monoclonal anti-HA-Tag (6E2) , Cell Signaling , Catalog no: #2367 , Lot no: 5, 1:500 dilution in western analyses;
Mouse monoclonal anti-HA (HA-7) , Sigma , Catalog no: H3663 , Lot no: 092M4827V, 1:200 dilution in immunofluoresence analyses ;
Rabbit monoclonal anti-JAK2 (D2E12) , Cell Signaling , Catalog no: #3230 , Lot no: 8, 1:1000 dilution in western analyses;
Rabbit monoclonal anti-Lamin B1 (D4Q4Z) , Cell Signaling , Catalog no: #12586 , Lot no: 2, 1:1000 dilution in western analyses;
Goat polyclonal anti-Lamin B (M-20) , Santa Cruz , Catalog no: sc-6217 , Lot no: J1712, 1:1000 dilution in western analyses;
Rabbit monoclonal p44/42 MAPK (Erk1/2), Cell Signaling, Catalog no: #9102, Lot no: 26, 1:2000 dilution in western analyses;
Rabbit polyclonal P-p44/42 MAPK (T202/Y204), Cell Signaling, Catalog no: #9101, Lot no: 29-30, 1:1000 dilution in western analyses;
Rabbit polyclonal anti-PDGFRα antibody, Millipore, Catalog no:07-276, Lot no: 2193158, 1:1000 dilution in western analyses;
Rabbit polyclonal anti-Myc-Tag , Cell Signaling , Catalog no: #2272 , Lot no: 6, 1:100 dilution in immunofluorescence analyses ;
Rabbit polyclonal anti-PEPP3 , Abcam , Catalog no: ab173483 , Lot no: GR201718-1, 1:1000 dilution in western analyses ;
Mouse monoclonal anti- POL II (8WG16) , Santa Cruz , Catalog no: sc-56767 , Lot no: A1415, 1:1000 dilution in western analyses ;
Rabbit monoclonal anti-Sodium Potassium ATPase (EP1845Y) , Abcam , Catalog no: ab76020 , Lot no: GR192449-15, 1:1000 dilution in western analyses;
Mouse monoclonal anti-Stat3 (124H6) (W), Cell Signaling, #9139, Lot no: 12, 1:1000 dilution in western analyses;
Rabbit monoclonal anti-Phospho-Stat3 (Tyr705) (D3A7) XP®,Cell Signaling, #9145, Lot no: 8, 1:500 dilution in western analyses;
Mouse monoclonal Phospho-Stat5 (Tyr694) (14H2) , Cell Signaling , Catalog no: #9356 , Lot no: 7, 1:500 dilution in western analyses ;
Rabbit polyclonal anti-Phospho-STAT5 (Tyr694) , Cell Signaling , Catalog no: #9351 , Lot no: 9, 1:500 dilution in western analyses;
Rabbit polyclonal anti-STAT5a (L-20) , Santa Cruz , Catalog no: sc-1081 , Lot no: 12613, 1:1000 dilution in western analyses, 1-2ug per 1mg of protein for immunoprecipitation ;
Mouse monoclonal anti-STAT5a (C-6) , Santa Cruz , Catalog no: sc-271542 , Lot no: #C1820, 1:1000 dilution in western analyses, 2ug per 1mg of protein for immunoprecipitation;
Rabbit polyclonal anti-STAT5a (Ab 780) , Sigma , Catalog no: SAB4300330 , Lot no: 511721049, 1ug per 1 mg of protein for immunoprecipitation ;
Rabbit polyclonal anti-STAT5a Prestige Antibodies , Sigma , Catalog no: HPA027873 , Lot no: R28826, 1:1000 dilution for western analyses, 2ug per 1mg of protein for immunoprecipitation;
Rabbit polyclonal anti-STAT5 (C-17) (recognizes STAT5b) , Santa Cruz , Catalog no: sc-835 , Lot no: L0413, 1:1000 dilution for western analyses, 1ug per 1mg of protein for immunoprecipitation;
Rabbit oligoclonal anti- STAT5b (13HCLC) , Thermo Fisher Scientific , Catalog no: 710139 , Lot no: 1040618B, 1ug per 1 mg of protein for immunoprecipitations ;
Rabbit polyclonal anti-STAT5b , R&D , Catalog no: AF1584 , Lot no: JFQ1517021, 2ug per 1 mg of protein for immunoprecipitation, 1:50 dilution for immunofluorescence analyses, 1:1000 dilution for western analyses;
Mouse monoclonal anti-STAT5B antibody (clone 2D1), Sigma, SAB1412214, Lot no: 4, 1:100 dilution for immunofluorescence analyses;
Rabbit polyclonal anti-TYK2 , Cell Signaling , Catalog no: #9312 , Lot no: 4, 1:1000 dilution for western analyses ;
Mouse monoclonal anti-p-Tyr (PY99)HRP , Santa Cruz , Catalog no: sc-7020 HRP , Lot no: F1909, 1:100 dilution for western analyses;
Goat anti-Mouse IgG (H+L) Highly Cross-Adsorbed Secondary Antibody, Alexa Fluor 555, Invitrogen, Catalog no: A-21424, Lot no: 1812159, 1:500 dilution for immunofluorescence analyses;
IRDye 680RD Donkey anti-Mouse IgG (H + L), LI-COR, Catalog no: 925-68072, Lot no: D10728-15, 1:10000 dilution for western analyses;
IRDye 800CW Donkey anti-Mouse IgG (H + L), LI-COR, Catalog no: 925-32212, Lot no: C90507-03, 1:10000 dilution for western analyses;
goat anti-mouse IgG-HRP, Santa Cruz, Catalog no: sc-2005, Lot no: #B2213, 1:10000 dilution for western analyses;
Goat anti-Rabbit IgG (H+L) Highly Cross-Adsorbed Secondary Antibody, Alexa Fluor 488, Invitrogen, Catalog no: A-11034, Lot no: 2018207, 1:500 dilution for immunofluorescence analyses;
Goat anti-Rabbit IgG (H+L) Highly Cross-Adsorbed Secondary Antibody, Alexa Fluor 555, Invitrogen, Catalog no: A-21429, Lot no: 2090567, 1:500 dilution for immunofluorescence analyses;
IRDye 680RD Donkey anti-Rabbit IgG (H + L), LI-COR, Catalog no: 926-68073, Lot no: D11102-14, 1:10000 dilution for western analyses;
IRDye 800CW Donkey anti-Rabbit IgG (H + L), LI-COR, Catalog no: 925-32213, Lot no: D11005-08, 1:10000 dilution for western analyses;
goat anti-rabbit IgG-HRP, Santa Cruz, Catalog no: sc-2004, Lot no: #D1216, 1:10000 dilution for western analyses;
Goat anti-Rabbit IgG (H+L) Cross-Adsorbed Secondary Antibody, HRP, Invitrogen, Catalog no: A16104, Lot no: 46-183-082415, 1:10000 dilution for western analyses;
Goat anti-rabbit IgG Abberior STAR 635, Abberior, Catalog no: 2-0012-002-7, Lot no: 14012016Cw, 1:100 dilution for immunofluorescence analyses;
Abberior STAR 580, goat anti-mouse IgG, Abberior, Catalog no: 2-0002-005-1, Lot no: 09072018CW/JR, 1:100 dilution for immunofluorescence analyses;

Validation

Mouse monoclonal anti-β-actin (AC-74) , Validated for Western analysis at manufacturer's website for human and mouse, in Fig. S8B the antibody produced a clear band of correct size in green monkey cells in Western analysis
Goat polyclonal anti-Actin (I-19) , Validated for Western analysis at manufacturer's website for human and mouse
Mouse monoclonal anti-β1-integrin (4B7R) , Validated for immunofluorescence at manufacturer's website for human, immunofluorescence staining pattern similar to the one in manufacturer's datasheet for green monkey cells

Mouse monoclonal anti-β1-integrin (K-20), Validated for mmunofluorescence at manufacturer's website for human, immunofluorescence staining pattern similar to the one in manufacturer's datasheet for green monkey cells

Rabbit monoclonal anti-β4-integrin (D8P6C), Validated for Western analysis at manufacturer's website for human, In Fig. 4D the antibody produced a clear band of correct size in green monkey cells

Rabbit polyclonal anti-β-Tubulin (H-235) , Validated for Western analysis at manufacturer's website for human and mouse

Mouse monoclonal anti-β-Tubulin (SAP.4G5) , Validated for Western analysis at manufacturer's website for human and mouse, In Fig. 4A,C and S7C the antibody produced a clear band of correct size in green monkey cells

Rabbit polyclonal EGF Receptor Antibody, Validated for Western analysis at manufacturer's website for human and mouse, In Fig. 5A produced a clear band of correct size in mouse cells

Rabbit monoclonal Phospho-EGF Receptor (Tyr1068) (D7A5), Validated for Western analysis at manufacturer's website for human and mouse, In Fig. 6A produced a clear band of correct size in mouse cells

Rabbit polyclonal ErbB2 Antibody (C-18), Validated for Western analysis at manufacturer's datasheet for human and mouse, In Fig. 5A produced a clear band of correct size in mouse cells

Rabbit monoclonal Phospho-HER2/ErbB2 (Tyr1248) Antibody, Validated for Western analysis at manufacturer's website for human and mouse, In Fig. 5A produced a clear band of correct size in mouse cells

Rabbit monoclonal anti-ErbB4 (E200) , Validated for immunoprecipitation, immunofluorescence and Western analysis at manufacturer's website for mouse and human

Mouse monoclonal anti-ErbB4 (HFR-1) , Validated for immunoprecipitation and Western analysis at manufacturer's website for mouse and human

Rabbit monoclonal anti- Phospho-HER4/ErbB4 (Tyr1284) (21A9) , Validated for Western analysis at manufacturer's website for human; in Fig 5A antibody shows reactivity against phosphorylated murine ErbB4 only after ErbB4 ligand treatment

Rabbit polyclonal anti-GFP , Validated for immunoprecipitation, immunofluorescence and Western analysis at manufacturer's website for transfected human cells, validated to recgonize all variants of Aequorea victoria GFP such as S65T-GFP, RS-GFP, YFP, CFP, RFP and EGFP.

Mouse monoclonal anti-HA-Tag (6E2) , Validated for Western analysis at manufacturer's website for mammalian cells transfected with the HA epitope tag only

Mouse monoclonal anti-HA (HA-7) , Validated for immunofluorescence at manufacturer's website for mammalian cells transfected with the HA epitope tag only

Rabbit monoclonal anti-JAK2 (D2E12) , Validated for Western analysis at manufacturer's website for human, in Fig. 1E,F the reactivity of the antibody is reduced in cells treated with JAK2 siRNA

Rabbit monoclonal anti-Lamin B1 (D4Q4Z) , Validated for Western analysis at manufacturer's website for human and mouse, in Fig. 4D, S6A the antibody produced a clear band of correct size in green monkey cells in Western analysis

Goat polyclonal anti-Lamin B (M-20) , Validated for Western analysis at manufacturer's website for human and mouse

Rabbit polyclonal anti-Myc-Tag , Validated for immunofluorescence at manufacturer's website for transfected cells

Rabbit monoclonal p44/42 MAPK (Erk1/2), Validated for Western analysis at manufacturer's website for mouse and human, , In Fig. 5A produced a clear band of correct size in mouse cells

Rabbit polyclonal P-p44/42 MAPK (T202/Y204), Validated for Western analysis at manufacturer's website for mouse and human, , In Fig. 5A produced a clear band of correct size in mouse cells

Rabbit polyclonal anti-PDGFRα antibody, Validated for Western analysis at manufacturer's website for mouse and human, In Fig. 5A produced a clear band of correct size in mouse cells

Rabbit polyclonal anti-PEPP3 , Validated for Western analysis at manufacturer's datasheet for human

Mouse monoclonal anti- POL II (8WG16) , Validated for Western analysis at manufacturer's datasheet for human and mouse

Rabbit monoclonal anti-Sodium Potassium ATPase (EP1845Y) , Validated for Western analysis and immunofluorescence at manufacturer's website for human and mouse

Mouse monoclonal anti-Stat3 (124H6) (W), Cell Signaling, #9139; Validated for western analysis at manufacture's sheet for human. In Fig. 4C produced a band of correct size in green monkey cells that was recognized also with a pSTAT3 antibody

Rabbit monoclonal anti-Phospho-Stat3 (Tyr705) (D3A7) XP®,Cell Signaling, #9145; Validated for western analysis at manufacture's sheet for human. In Fig. 4C produced a band of correct size in green monkey cells that was recognized also with a STAT3 antibody

Mouse monoclonal Phospho-Stat5 (Tyr694) (14H2) , Validated for Western analysis at manufacturer's website for human and mouse, In Fig. 3D the antibody recognized a clear band of correct size that was enriched by immunoprecipitation with a STAT5b antibody

Rabbit polyclonal anti-Phospho-STAT5 (Tyr694) , Validated for Western analysis at manufacturer's website for human and mouse, In Fig. 3D, 4A, 4C, S8C and S10A-B the antibody recognized a clear band of correct size that was enriched by immunoprecipitation with STAT5a and STAT5b antibodies and recognized by other STAT5 antibodies in Western analysis in green monkey cell samples

Rabbit polyclonal anti-STAT5a (L-20) , Validated for Western analysis, immunoprecipitation and immunofluorescence at manufacturer's datasheet for human and mouse

Mouse monoclonal anti-STAT5a (C-6) , Validated for Western analysis at manufacturer's website for human and mouse, was validated in house for immunoprecipitation in green monkey samples (Fig. S10A-C) by detecting enrichment of a band that was detected with other STAT5a and pSTAT5 antibodies in Western analysis

Rabbit polyclonal anti-STAT5a (Ab 780) , Validated for Western analysis at manufacturer's website for human, was validated in house for immunoprecipitation in green monkey samples (Fig. S8C) by detecting enrichment of a band that was detected with other STAT5a and pSTAT5 antibodies in Western analysis

Rabbit polyclonal anti-STAT5a Prestige Antibodies , Validated for Western analysis at manufacturer's website for human, was validated in house for immunoprecipitation in and green monkey samples (Fig. 4A) by detecting enrichment of a band that was detected with other STAT5a and pSTAT5 antibodies in Western analysis (more data available upon request)

Rabbit polyclonal anti-STAT5 (C-17) (recognizes STAT5b) , Validated for Western analysis, immunoprecipitation and immunofluorescence at manufacturer's datasheet for human and mouse, was validated in house with Western analysis of cells treated with STAT5a- and STAT5b-specific siRNAs to selectively recognize STAT5b (data available upon request), in Fig. 2B,4C,

S6C, S10B the antibody recognized a band of correct size that was enriched by immunoprecipitation with other STAT5b antibodies in green monkey cells in Western analysis
Rabbit oligoclonal anti- STAT5b (13HCLC) , Validated for Western analysis at manufacturer's datasheet for human
Rabbit polyclonal anti-STAT5b , Validated for Western analysis, immunoprecipitation and immunofluorescence at manufacturer's datasheet for human and mouse, In Fig. S8C, S6G, 4C immunoprecipitation with the antibody in green monkey cells enriched a band of correct size that was recognized by other STAT5b and pSTAT5 antibodies
Mouse monoclonal anti-STAT5B antibody (clone 2D1), Sigma, SAB1412214; Validated for immunofluorescence at manufacturer's datasheet for human
Rabbit polyclonal anti-TYK2 , Validated for Western analysis at manufacturer's website for human, in Fig. 1E,F the reactivity of the antibody is reduced in cells treated with TYK2 siRNA
Mouse monoclonal anti-p-Tyr (PY99)HRP , Validated for Western analysis at manufacturer's website for human and mouse

Eukaryotic cell lines

Policy information about cell lines

Cell line source(s)	MCF7, European Collection of Cell Cultures ;<br>HC11, a kind gift from Dr. Lars-Arne Haldosén, Karolinska Institutet, Huddinge, Sweden, original commercial source: ATCC ;<br>COS-7, a kind gift from Dr. Lea Sistonen, Turku Bioscience Centre, University of Turku, Turku, Finland, original commercial source: ATCC ;<br>MDA-MB-468, a kind gift from Dr. Johanna Ivaska, Turku Bioscience Centre, University of Turku, Turku, Finland, original commercial source: ATCC;<br>Phoenix Ampho HEK293 cells, ATCC
Authentication	None of the cell lines used were authenticated
Mycoplasma contamination	Mycoplasma contamination was sporadically tested. Cells that tested positive for mycoplasma contamination were disposed.
Commonly misidentified lines<br>(See ICLAC register)	No commonly misidentified cell lines were used
